# Injectable Nanorobot-Hydrogel Superstructure for Hemostasis and Anticancer Therapy of Spinal Metastasis

**DOI:** 10.1007/s40820-024-01469-3

**Published:** 2024-08-01

**Authors:** Qing Chen, Miao Yan, Annan Hu, Bing Liang, Hongwei Lu, Lei Zhou, Yiqun Ma, Chao Jia, Dihan Su, Biao Kong, Wei Hong, Libo Jiang, Jian Dong

**Affiliations:** 1 Department of Orthopaedic Surgery, Zhongshan Hospital, Fudan University, Shanghai, 200032 People’s Republic of China; 2 Department of Chemistry, Fudan University, Shanghai, 200438 People’s Republic of China; 3 Department of Surgery, Yong Loo Lin School of Medicine, National University of Singapore, 1E Kent Ridge Road, Singapore, 119228 Singapore; 4 Department of Geriatrics and Gerontology, Huadong Hospital, Fudan University, Shanghai, 200438 People’s Republic of China; 5 State Key Laboratory of Molecular Engineering of Polymers, Fudan University, Shanghai, 200438 People’s Republic of China; 6 Department of Orthopaedic Surgery Zhongshan Hospital Wusong Branch, Fudan University, Shanghai, 200940 People’s Republic of China

**Keywords:** Silk fibroin, Nanofibril hydrogels, Nanorobots, Thrombin, Spinal metastasis

## Abstract

Surgery remains the standard treatment for spinal metastasis. However, uncontrolled intraoperative bleeding poses a significant challenge for adequate surgical resection and compromises surgical outcomes. In this study, we develop a thrombin (Thr)-loaded nanorobot-hydrogel hybrid superstructure by incorporating nanorobots into regenerated silk fibroin nanofibril hydrogels. This superstructure with superior thixotropic properties is injected percutaneously and dispersed into the spinal metastasis of hepatocellular carcinoma (HCC) with easy bleeding characteristics, before spinal surgery in a mouse model. Under near-infrared irradiation, the self-motile nanorobots penetrate into the deep spinal tumor, releasing Thr in a controlled manner. Thr-induced thrombosis effectively blocks the tumor vasculature and reduces bleeding, inhibiting tumor growth and postoperative recurrence with Au nanorod-mediated photothermal therapy. Our minimally invasive treatment platform provides a novel preoperative therapeutic strategy for HCC spinal metastasis effectively controlling intraoperative bleeding and tumor growth, with potentially reduced surgical complications and enhanced operative outcomes.

**Supplementary Information:**

The online version contains supplementary material available at 10.1007/s40820-024-01469-3.

## Highlights

- Site-selective superassembly was used to synthesize a nanorobot with bionic rod-shaped head/hollow tail structure, exhibiting strong photothermal effect, high drug loading capacity, precise and controlled drug release, and excellent motility.
- Injectable regenerated silk fibroin (RSF) nanofibril hydrogels were developed via simple sonication, offering significant production, structural, and performance advantages.
- Nanorobot/thrombin (Thr)/RSF nanofibril hydrogels reduced intraoperative bleeding of hepatocellular carcinoma (HCC) spinal metastasis, provided starvation embolization, and prevented post-surgery recurrence.
- Nanorobot/Thr/RSF nanofibril hydrogels inhibited blood supply to residual tumors and reduced neovascularization, inhibiting post-surgery HCC spinal metastasis recurrence.

**Supplementary Information:**

The online version contains supplementary material available at 10.1007/s40820-024-01469-3.

## Graphical abstract

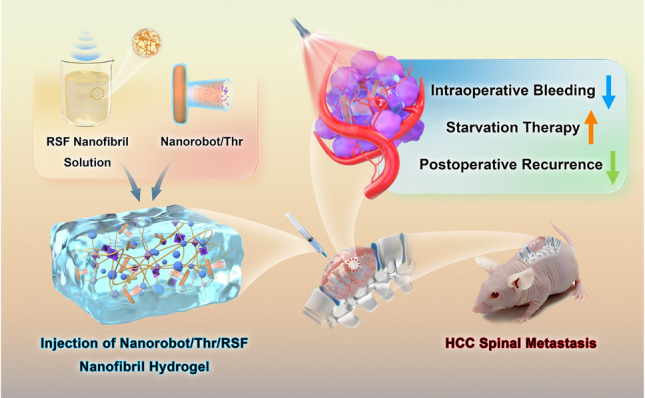

## Introduction

Spinal metastasis poses a significant clinical challenge in modern oncology [1, 2]. Currently, the treatment of spinal metastasis relies on surgery and radiotherapy [3, 4]. However, uncontrolled intraoperative hemorrhage poses a significant challenge in spinal metastatic tumor surgery, resulting in compromised surgical visualization, prolonged operative duration, incomplete tumor excision, heightened risk of spinal cord injury, and potential jeopardy to the lives of patients [5, 6]. Furthermore, excessive bleeding during the procedure may impede complete tumor removal and contribute to postoperative recurrence [7]. Studies show that the average perioperative blood loss volume for spinal metastasis is 2180 mL [8], and the intraoperative blood loss and postoperative recurrence rate are higher for primary tumors originating from hepatocellular, renal, or thyroid carcinomas [9]. Therefore, effective control of intraoperative blood loss and reduction of postoperative recurrence are key factors in successful spinal tumor surgery.

Preoperative embolization of spinal metastasis is currently considered the optimal strategy for preventing intraoperative bleeding; it is widely employed to diminish tumor blood supply and has demonstrated efficacy in reducing intraoperative blood loss [10]. Nevertheless, there is ongoing discourse regarding the utilization of preoperative embolization for spinal metastasis. Preoperative embolization carries the risk of nerve injury, spinal cord ischemia, lower extremity paralysis, and cutaneous muscle necrosis, increasing perioperative complications [11, 12]. For economic and safety reasons, in situ injection of embolic agents is an alternative treatment method that not only reduces the cumbersome steps of preoperative embolization compared with percutaneous arterial catheter embolization but also avoids the above operational complications. In clinical practice, thrombin (Thr) is commonly used to achieve local hemostasis during surgery. However, Thr can cause nonspecific embolization after entering the body and contacting blood, resulting in serious toxic side effects. The direct local injection of Thr is ineffective for hemostasis and can only perform surface hemostasis without accessing deeper bleeding sites [13]. Therefore, there is an urgent need to develop new hemostatic materials that can deliver Thr to deep bleeding sites and accelerate the hemostatic process.

Insufficient penetration of tumors and uncontrolled drug release by conventional nanoparticles remain significant challenges [14]. Recently, self-propelled micro/nanorobots that possess active motility to facilitate deep tumor penetration have garnered attention [15–17]. However, the use of drug-carrying nanoparticles and nanorobots alone has common drawbacks. For example, their systemic administration may result in organ-specific toxicity. Even when administered locally, they are susceptible to cell-mediated transport away from the injection site or interstitial leakage within tissues [18, 19]. Hydrogel-loaded drugs or nanoparticles can well address the above challenges, but still suffer from problems such as uncontrolled release and diffusion limitations [20]. Therefore, nanorobots require specific properties of hydrogel carriers to locally control the release and avoid the diffusion of nanorobots into normal tissues, which ultimately performs optimal biological functions. Regenerated silk fibroin (RSF), a naturally derived biopolymer extracted from silkworm cocoons, exhibits excellent biocompatibility, degradability, propensity for self-assembly, adhesion, and hemostatic properties [21, 22]. Injectable RSF hydrogels are formed through physical and chemical methods to induce gelation, albeit with a slow process, however, it commonly needs several minutes or more [23]. Subsequently, upon injection, RSF undergoes non-covalent or covalent in situ cross-linking reactions to transform from a solution into a hydrogel state. Injectable RSF nanofibril hydrogels have better thixotropic properties, higher shear-thinning performance, higher loading capacity, better biocompatibility, and a more homogeneous network composed of nanofibrils with fast gel-sol–gel transition [24, 25]. Therefore, the synthesis of nanorobot-hydrogel superstructures can effectively address these challenges including low local enrichment of nanoparticles, poor infiltration in the tumor, and the complexity of the current preparation process of RSF nanofibril hydrogels. Until now, there are no reports on the preparation, hemostatic properties, and anticancer therapy of injectable hybrid nanorobot-RSF nanofibril hydrogels.

**Fig. 1:**
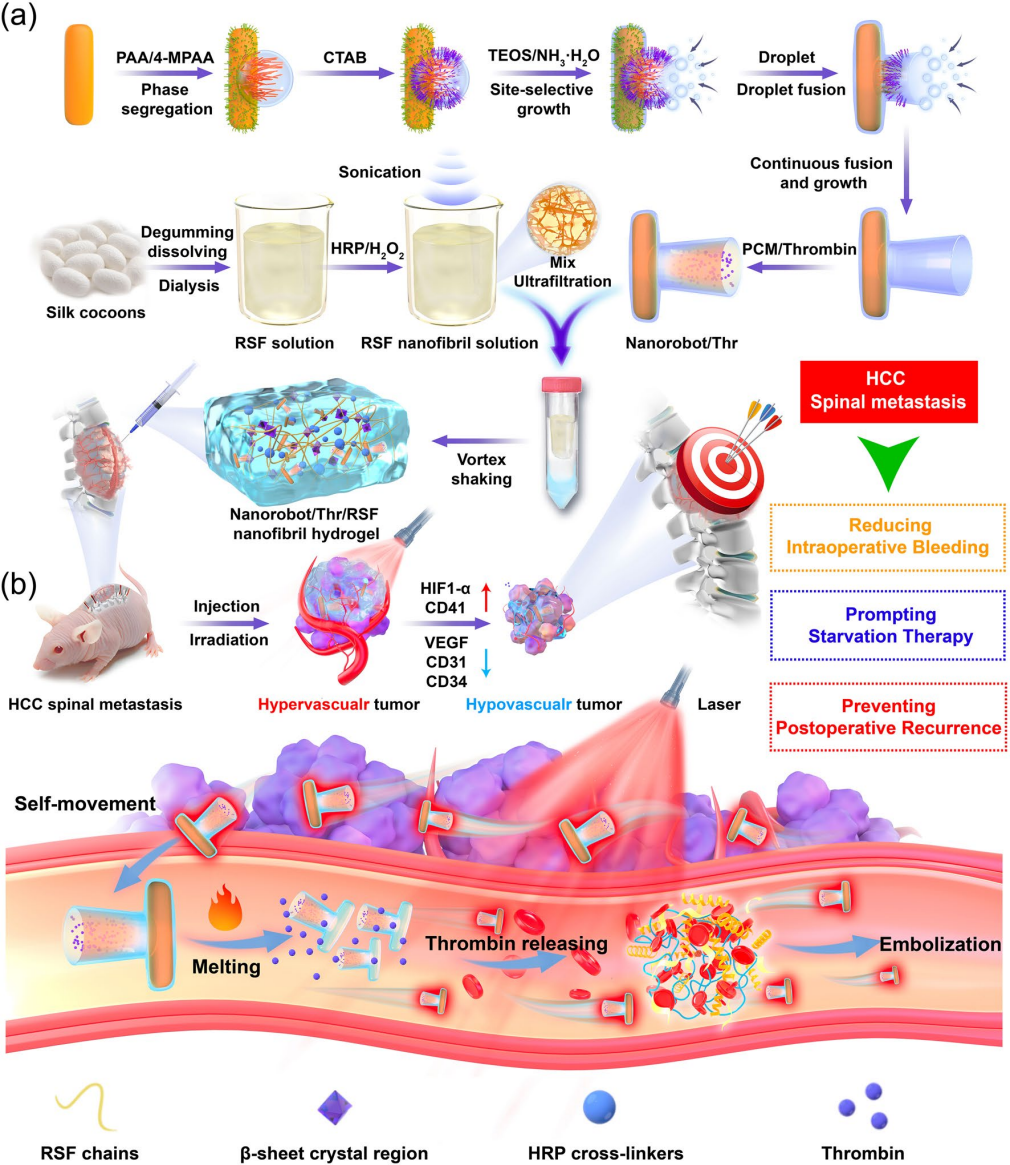
Mechanism of action of Nanorobot/Thr/RSF nanofibril hydrogels in the treatment of HCC spinal metastasis. **a** Super-assembly of nanorobots, the preparation of RSF nanofibril solutions, and Nanorobots/Thr/RSF nanofibril hydrogels. **b** The Nanorobot/Thr/RSF nanofibril hydrogels for reducing intraoperative bleeding in HCC spinal metastasis, providing starvation embolization therapy, and preventing postoperative recurrence of HCC spinal metastasis

Here, we developed the novel injectable RSF nanofibril hydrogels with nanofibril network, excellent thixotropy, higher shear-thinning performance, higher loading capacity, and good biocompatibility. We simultaneously incorporated Thr-loaded nanorobots into the nanofibril structure of the hydrogels to obtain the nanorobot-hydrogel hybrid superstructure using a simple two-step sonication and ultrafiltration process. The superior thixotropic properties of the RSF nanofibril hydrogels allow for better coverage and penetration of hepatocellular carcinoma (HCC) spinal metastasis tumor tissue. The active motion capability of nanorobot further facilitates deep tumor penetration. Additionally, nanorobot enabled the co-encapsulation of phase change material (PCM, PCM with huge latent heat of phase change have been used as safe drug carriers for controlled release with reversible solid–liquid phase change at specific temperatures) inside large cavity and controllable delivery of Thr triggered by 980 nm near infrared (NIR) irradiation to raise the temperature above PCM melting point due to excellent photothermal effect of AuNR [26]. Precise and rapid Thr release results in thrombus formation in HCC spinal metastasis. The resulting thrombus cuts-off blood and nutrient supply to tumor cells, transforming the HCC spinal metastasis from a blood-rich to blood-poor state, while inducing apoptosis and necrosis of tumor cells and reducing the secretion of angiogenesis-promoting signaling molecules (Fig. 1). These integrated and synergistic treatment strategies create ideal conditions for spinal metastasis surgery using minimally invasive techniques, besides reducing the risk of bleeding during surgery. Simultaneously, more comprehensive anti-cancer therapies may be provided to patients with spinal metastasis, bringing innovative possibilities to perioperative treatments.

## Methods

### Materials

Silver nitrate (AgNO_3_), Gold (III) chloride trihydrate (HAuCl_4_, ≥ 99.9% trace metals basis), and L-ascorbic acid (LAA) were supplied by Alfa Aesar (China); sodium citrate tribasic dihydrate (99.0%), 4-mercaptophenylacetic acid (4-MPAA, 97%), tetraethyl orthosilicate (TEOS) (≥ 99.0%), hexadecyltrimethylammonium bromide (CTAB) were purchased from Sigma-Aldrich (USA); sodium oleate (> 97.0%) were purchased from Macklin (China); Poly (acrylic acid) (PAA) (average M_w_ = 5000) was purchased from Polymer Source, Inc (USA); 2-Propanol (HPLC grade) and ammonium hydroxide (28.0%–30.0%) were purchased from Macklin. Lauric acid (97%) and stearic acid (95%) were obtained from Alfa Aesar.

### Synthesis of Monodisperse Au Nnanorods

Au nanorods (AuNRs) were synthesized according to a previously reported method [27].

### Synthesis of AuNR-Silica Nanoparticles (AuNR-Silica NPs)

AuNR solution (4 mL) was centrifuged and washed twice with water to remove excess CTAB. The concentrated AuNRs were then redispersed in 400 μL water. The AuNR aqueous solution obtained was added dropwise to a water/IPA mixture containing 2.5 mL of 2-propanol, 25 μL of 4-MPAA (25 mM in ethanol), and 25 μL of PAA (0.65 mM in H_2_O) under vigorous stirring for 30 min. Next, 600 μL of CTAB (0.0015 g mL^−1^ in H_2_O), 2 μL of TEOS, and 90 μL of ammonium hydroxide solution (pH = 10.98) were added with slow stirring (330 rpm). After 6 h of reaction, the products were collected by centrifugation at 2500 rpm for 7 min and washed with ethanol.

### Synthesis of AuNR-Silica/Thr Nanoparticles

1-Hexadecanol and oleic acid were weighed in a mass ratio of 3.5:1 and dissolved in absolute ethanol. The mixture was then ultrasonically mixed and stored at 4 °C for subsequent use. Afterward, PCM was dissolved in 1 mL ethanol solution containing 20 mg of AuNR-Silica NPs and Thr. The obtained suspension was then kept in a vacuum oven at 50 °C for 10 min to remove the air trapped in the hollow interior of the AuNR-Silica NPs. Finally, the AuNR-Silica NPs loaded with the PCM and Thr suspension were collected by centrifugation at 300 × g for 7 min, and the supernatant was removed. The excess PCM and Thr were removed after two additional rounds of washing with ethanol. Subsequently, the AuNR-Silica NPs were immediately cooled in an ice bath to obtain the Thr-loaded AuNR-Silica NPs.

### Synthesis of AuNR-Silica/Cy5.5 NPs

Initially, Cy5.5 was conjugated with 3-Aminopropyltriethoxysilane (APTES). To achieve this, 1 mg of Cy5.5-NHS and 10 μL of APTES were dissolved in 500 μL of DMSO. This mixture was stirred for 24 h at room temperature in a dark environment. Subsequently, 50 μL of the Cy5.5-APTES solution was added to 2 mL of an ethanol solution containing the nanotadpoles. This new mixture was then stirred for an additional 12 h in the dark. As a result, Cy5.5 was successfully conjugated with the nanotadpoles.

### Calculating and Analyzing the Mean Square Displacement for Nanorobots

The methods for recording and tracking the motion of the Nanorobots, as well as calculating and analyzing the mean-square displacements, are referenced from previous reports [28].

### Preparation of RSF Solution

Initially, Bombyx mori silkworm cocoons underwent two rounds of degumming at 100 °C for 30 min, employing a Na_2_CO_3_ solution (0.02 M). Next, the cocoons were meticulously rinsed with distilled water and subsequently dried at 40 °C. Subsequently, the degummed silk was immersed in a LiBr solution (9.3 M) and incubated at 60 °C for 1 h. Dialysis was performed at room temperature using a dialysis membrane with a molecular weight cut-off of 14,000, over at least 72 h, to eliminate residual salt. This extensive dialysis yielded an aqueous RSF solution with a concentration of 5 wt%, which was further diluted to 0.75 wt%.

### Preparation of RSF Nanofibril Solutions by Sonication

To promote nanofibril formation, 0.75 wt% RSF solution (with or without HRP 900 U/mL^−1^, H_2_O_2_ 0.5 v/v%, 200 μL) was sonicated using an Ultrasonic Cell Disruptor (VOSHIN-150XB, Voshin, China). Subsequent sonication experiments were carried out at 50% duty cycle (repeated cycles of 0.5 s sonication and 0.5 s shutdown) to minimize temperature variations and at an output power of 300 W, for different durations (5, 10, 15, and 30 min), to promote nanofibril formation and ultimately, to obtain RSF solution containing nanofibrils. The entire process was performed in an ice-water bath.

### Preparation of Injectable AuNR-Silica NP/RSF Nanofibril Hydrogels

Before preparing RSF nanofibril hydrogels, AuNR-Silica NP solution was mixed into the RSF nanofibril solutions. The resulting solution was centrifuged at 5000 rpm for 60 min. A uniform distribution of the AuNR-Silica NPs (100 μg mL^−1^) in the RSF nanofibril hydrogels were ensured by vortex shaking for 10 min.

#### Characterization of the RSF Solution and AuNR-Silica NP/RSF Nanofibril Hydrogels

The microstructures of the RSF solution and Nanorobot/RSF nanofibril hydrogels were observed using X-ray diffraction (XRD) (Bruker D8 ADVANCE and DAVINCI.DE-SIGN X-ray powder diffractometer, Germany), scanning electron microscopy-elemental energy spectroscopy (SEM–EDS, Sigma 300, Zeiss, Jena, Germany), and cryo-scanning electron microscopy (cryo-SEM) (Hitachi S4800, Japan). The fourier transform Infrared (FTIR) spectra of the lyophilized RSF solution and RSF nanofibril hydrogels were recorded using an FTIR spectrometer (Nicolet 6700, Thermo Fisher, USA) at wavelengths 4000 to 400 cm^−1^.

#### Rheological Test

Rheological assessments were conducted under controlled conditions at a temperature of 37 ± 1 °C using a Physica MCR 301 rheometer (Anton Paar GmbH, Austria), configured with 25 mm parallel-plate geometry featuring a 1 mm gap distance. A volume of 1 mL of the RSF nanofibril hydrogels and Nanorobot/RSF nanofibril hydrogels was deposited onto the lower plate and a layer of low-density mineral oil was applied around the hydrogels to mitigate water evaporation. For dynamic strain scanning tests, the strain range was applied from 0.1% to 1000%. Frequency sweeps were performed across a range of 0.1 to 10 Hz while maintaining a constant strain of 0.1%. For shear recovery analysis, the hydrogels were initially subjected to a strain of 0.1%, which was elevated to 5000% and reverted to the original 0.1%. This cyclic procedure was repeated multiple times. Finally, shear-rate scanning tests were performed on the hydrogels.

#### In Vitro Degradation of RSF and Nanorobot/RSF Nanofibril Hydrogels

The degradation of RSF nanofibril hydrogels was tested in vitro. The RSF and Nanorobot/RSF nanofibril hydrogels were immersed in phosphate buffer saline (PBS, HyClone, USA) solution containing 2 U mL^−1^ protease XIV (Sigma-Aldrich, USA) at 37 °C, and the RSF and Nanorobot/RSF nanofibril hydrogels were removed after 7 days to measure the loss in mass. Hydrogel mass retention (W_R_) was calculated as W_R_ = W_1_/W_0_ × 100%, where W_0_ is the weight of the hydrogel before immersion and W_1_ is the weight of the hydrogels after immersion.

#### Photothermal Conversion Efficiency

The photothermal effect of Nanorobot/RSF nanofibril hydrogels were experimentally investigated using a 980 nm laser system. First, RSF nanofibril hydrogels containing Nanorobot (100 μg mL^−1^) were prepared. The Nanorobot/RSF nanofibril hydrogels (500 μL) were placed in 6-well plates. These were irradiated with a 980 nm laser (2.0 W cm^−2^) for 10 min. During irradiation, the temperature was recorded every 30 s with a thermal imaging camera. The photothermal conversion efficiency of the Nanorobot/RSF nanofibril hydrogels were calculated based on previous reports [29–31].

#### In Vitro Thr Release Activity

The Thr activity was measured using a Thr Activity Fluorometric Assay Kit. Briefly, Nanorobot/Thr/RSF nanofibril hydrogels were irradiated with a 980 nm laser for 10 min to rapidly release the Thr loaded in the nanofibril hydrogels. When the substrate was used to assay Thr activity, the concentration of Thr was 90 nM, and the reaction volume was 100 μL. Thr protein hydrolysis cleaves synthetic substrates to release fluorophores that can be easily quantified using fluorescence. The fluorescence intensity was measured using a fluorescence spectrophotometer (FS5, Edinburgh Instruments, UK).

#### Cell Lines

The MHCC-97H, 786-O, FTC-133, AML-12, MHCC-97H-Luc, 786-O-Luc, and FTC-133-Luc cell lines were obtained from the Cell Bank of the Chinese Academy of Sciences in Shanghai, China. Cells were cultured in Dulbecco’s modified Eagle’s medium (DMEM, HyClone, USA) and DMEM/F12 (HyClone, USA) supplemented with 10% heat-inactivated fetal bovine serum (FBS, BI, Israel) and 1% penicillin/streptomycin (HyClone, USA). The culture medium was refreshed every 2 days, and cells were maintained in a humidified incubator at 37 °C with 5% CO_2_.

#### Cytotoxicity and Live/Dead Cell Assays

The cytocompatibility of the Nanorobot/Thr and Nanorobot/Thr/RSF nanofibril hydrogels was assessed in MHCC-97H cells using a Cell Counting Kit-8 (CCK-8, Dojindo, Japan). MHCC-97H cells were seeded into 96-well plates and partitioned into three replicate wells. Following overnight incubation, cytotoxicity was evaluated by exposing the cells to AuNR-Silica NP/Thr and AuNR-Silica NP/Thr/RSF nanofibril hydrogels for 24 and 48 h, respectively. Subsequently, 10% CCK-8 reagent was introduced and incubated at 37 °C for 2 h; absorbance at 450 nm was measured using a multi-plate instrument (FlexStation3, Molecular Devices, USA). The experimental procedure was repeated in 786-O and FTC-133 tumor cells, and in AML-12 normal cells. Further demonstration of cytotoxicity of the AuNR-Silica NP/Thr/RSF nanofibril hydrogels on MHCC-97H cells was achieved through fluorescence staining using a live/dead cytotoxicity kit (KeyGEN BioTECH, China). Post-incubation with AuNR-Silica NP/Thr and AuNR-Silica NP/Thr/RSF nanofibril hydrogels, MHCC-97H cells underwent staining with PI (red) and calcein AM (green) solutions at 37 °C and 5% CO_2_ for 40 min. Live/dead MHCC-97H cells were examined under a fluorescence microscope (BX53, Olympus), and quantified using Image J software 2.3.0. The same experimental protocol was applied to the 786-O and FTC-133 tumor cells, and AML-12 normal cells.

#### DNA Damage Assay

γ-H2AX staining was used to observe the DNA damage effects of different treatment groups on MHCC-97H cells. Briefly, MHCC-97H cells (5 × 10^5^ cells per well) were cultured in 12-well plates for 24 h. The treatment groups were as follows: Control, RSF, AuNR-Silica NP/RSF, Nanorobot/RSF + Laser (980 nm laser, 2.0 W cm^−2^ for 10 min), AuNR-Silica NP/Thr/RSF, and Nanorobot/Thr/RSF + Laser (980 nm laser, 2.0 W cm^−2^ for 10 min). Staining was detected using the γ-H2AX kit (Beyotime, China) and finally observed under a confocal laser scanning microscope (CLSM).

#### Construction of a Spinal Tumor Mouse Model

Six-week-old nude mice were procured from Jiesijie Laboratory Animal Co., LTD (Shanghai, China). All animal procedures adhered to the guidelines of the Animal Ethics Committee of the Zhongshan Hospital, Fudan University. To establish a spinal tumor model in mice, animals were placed in the prone position to expose the subcutaneous area, followed by disinfection with 75% alcohol. A mark denoting the midpoint of the line connecting the spine and iliac crest served as the puncture entry point. MHCC-97H cells, resuspended in PBS and thoroughly mixed, were injected slowly (25 μL; density 1 × 10^6^ cells mL^−1^) into the vertebral body using a needle inserted from the designated puncture point. Careful rotation of the needle ensured proper insertion into the vertebral bodies of the mice (confirmed by the distinct resistance at the needle tip). This approach was consistently applied to 786-O and FTC-133 tumor cells.

#### In Vivo Hemostatic and Intraoperative Hemorrhagic Reduction Properties of Nanorobot/Thr/RSF Nanofibril Hydrogels

In vivo hemostatic capacity was assessed using a mouse tail amputation and hepatectomy model. Following the mouse tail amputation, the wound was exposed to air for 15 s to maintain normal blood loss. Subsequently, Nanorobot/Thr/RSF nanofibril hydrogels were injected into the wound and irradiated with a 980 nm laser (2.0 W cm^−2^) to evaluate their hemostatic effect while recording the bleeding amount and time for hemostasis. For liver exposure in mice, a small portion of the liver was excised, and the hydrogel was injected at the incision site, followed by irradiation with a 980 nm laser (2.0 W cm^−2^) to assess its hemostatic effect while measuring the bleeding amount and time. To establish a mouse HCC spinal metastasis model, Nanorobot/Thr/RSF nanofibril hydrogels were injected, followed by irradiation with a 980 nm laser (2.0 W cm^−2^). After 24 h, the skin incision allowed tumor exposure in the spinal region where visible tumors were excised while observing the hemorrhage and recording the bleeding amount.

#### In Vivo Photothermal Effect

To further investigate the photothermal effect of Nanorobot/RSF nanofibril hydrogel in vivo, RSF nanofibril hydrogels and Nanorobot/RSF nanofibril hydrogels were injected into the HCC spinal metastasis model, respectively. Then the tumor was irradiated with a 980 nm (2.0 W cm^−2^) laser for 10 min, and the temperature change was recorded with a thermal imaging camera.

#### In Vivo Treatment of HCC Spinal Metastasis

After successful establishment of the spinal metastasis mouse model, the mice with HCC spinal metastasis were divided into the following five groups, each containing five mice: Saline, RSF, Nanorobot/RSF + Laser (980 nm laser, 2.0 W cm^−2^ for 10 min), AuNR-Silica NP/Thr/RSF, and Nanorobot/Thr/RSF + Laser (980 nm laser, 2.0 W cm^−2^ for 10 min). Body weight, paralysis rate, and tumor growth in the nude mice were observed during treatment. Upon completion of the treatment regimen, HCC spinal tumors were excised to measure their volumes and weights for assessment of treatment efficacy across various groups. To comprehensively evaluate both the tumor response and bone growth at the spinal tumor site, the excised spine was scanned and analyzed using a SkyScan micro-CT scanner (Bruker, Germany). Following 3D reconstruction using ParaVision software 4.1 (Aartselaar SkyScan, Belgium) and NRecon software 2.1 (Aartselaar SkyScan, Belgium), regions of interest were identified at the spinal tumor sites for subsequent qualitative and quantitative analysis. Parameters such as bone mineral density (BMD), bone volume fraction (BV/TV), trabecular thickness (Tb.Th), and trabecular separation (Tb.Sp) of the spinal tumor specimens were quantified using the CTAn software 1.15 (Aartselaar SkyScan, Belgium).

#### Resection Model of HCC Spinal Metastasis

To create the HCC spinal metastasis resection model, the HCC spinal metastasis model was first established as described above [28]. On day 14 after inoculation with tumor cells, mice carrying tumors underwent surgical resection of HCC metastasis, and the Nanorobot/Thr/RSF nanofibril hydrogels were injected into the tumor resection cavity of each nude mouse and subjected to laser irradiation. Thirty-five days after inoculation, each group of mice underwent examinations by bioluminescence imaging, tumor measurement, and histological analysis of recurrent HCC spinal metastases.

#### Immunofluorescence Studies

The tumor specimens were fixed in paraformaldehyde and embedded in paraffin. Tumor tissue sections measured 10 µm in thickness. In the tumor proliferation assay, Ki67 staining of tumor tissue sections was performed using a Ki67 antibody detection kit (Proteintech, USA) following the manufacturer's protocols. For the apoptosis assay, frozen spinal tumor sections were incubated with TUNEL solution (Servicebio, China) following the manufacturer’s protocol, with nuclei staining achieved using DAPI (Beyotime, China). Additionally, spinal metastasis of HCC was collected and subjected to immunostaining with antibodies targeting CD41, HIF-1, CD31, CD34, and VEGF for subsequent analysis. Comprehensive imaging of all tumor-sectioned tissues was performed using a CLSM.

#### Western Blotting

Mouse spinal tumor specimens were prepared as protein samples. Protein samples from each group were separated by SDS-PAGE, transferred to PVDF membranes, and incubated with primary antibodies (Anti-HIF-1 alpha antibody, Abcam; Anti Caspase-3 antibody, Abcam; Anti-P53 antibody, Abcam; Anti-VEGF antibody, Abcam, Anti-CD31 antibody, Abcam; Anti-CD34 antibody, Abcam; Anti-beta Actin Antibody, Abcam) overnight. The samples were then incubated with the corresponding secondary antibodies and exposed to ECL reagent (Thermo Fisher Scientific, USA). Quantitative analysis was performed using the ImageJ software 2.3.0.

#### Histological Analysis and Blood Chemistry

To investigate the biosafety of the Nanorobot/Thr/RSF nanofibril hydrogel injections in vivo, pivotal organs (heart, liver, spleen, lung, and kidney) were harvested post-treatment and preserved in paraformaldehyde. After fixation, the tissues were embedded in paraffin and sectioned for hematoxylin and eosin staining (H&E). Blood specimens were promptly obtained for comprehensive biochemical analyses, including routine blood, liver function, and kidney function assessments.

#### Statistical Analysis

Data from more than three replicates are expressed as mean ± standard deviation (SD). Unpaired Student’s t test was used to test for differences between groups. All data were statistically analyzed using GraphPad Prism 8, and *P* < 0.05 was considered statistically significant.

## Results and Discussion

### Characterization of AuNR-Silica Nanorobots

**Fig. 2:**
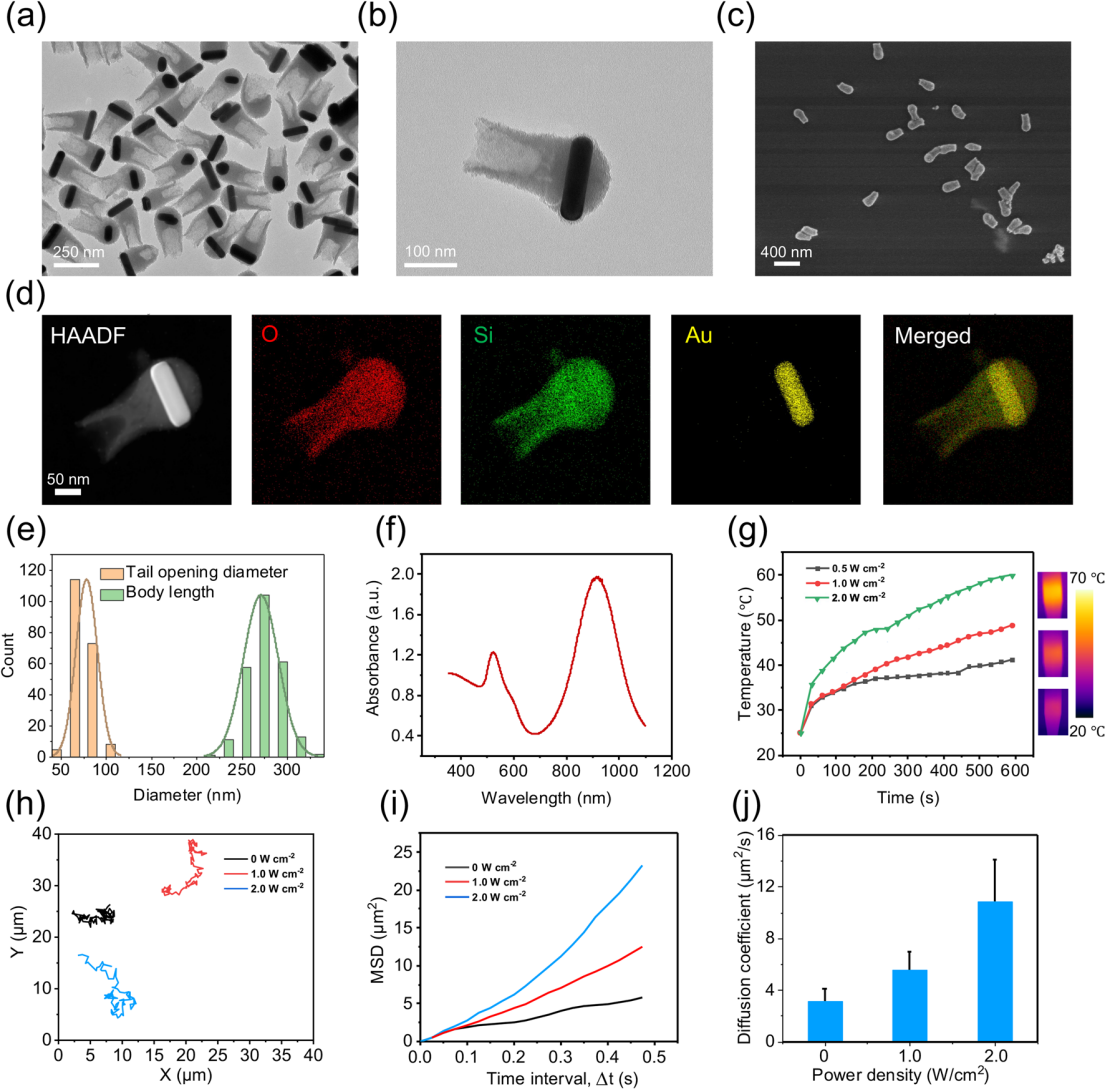
AuNR-Silica nanorobot fabrication and characterization. **a** Low-resolution TEM image of the as-synthesized nanotadpoles. **b** Magnified TEM image showing an individual nanotadpole. **c** SEM image of nanotadpoles. **d** Elemental mapping of the nanotadpole. **e** Body length distribution and tail opening diameter of a nanotadpole determined by TEM (200 nanoparticles analyzed). **f** UV–vis-NIR spectra recorded from aqueous suspensions of the nanotadpoles. **g** Photothermal effect of nanotadpoles in aqueous solution upon 980 nm laser irradiation under different power densities for 10 min. **h–i** Trajectories and mean square displacement (MSD) of PCM/Nanorobots irradiated with different NIR power densities. **j** The average diffusion coefficient (D) values. Experimental data are expressed as mean ± s.d. (n = 5)

AuNRs with an average length of 150 ± 7 nm and width of 40 ± 8 nm were used as the initial seeds (Fig. S1). The AuNRs-Silica nanotadpoles were synthesized using a previous method with slight modifications [28]. Briefly, the AuNRs were added to an isopropyl alcohol (IPA)-H_2_O mixture (2.5:1 V/V) containing (4-mercaptophenylacetic acid, 4-MPAA) and polyacrylic acid (PAA, M.W. = 5500), followed by stirring at room temperature for 30 min. Then, CTAB, TEOS, and ammonium hydroxide were added to trigger silica growth. After 6 h of reaction, AuNRs-Silica nanotadpoles were formed. Transmission electron microscopy (TEM) and scanning electron microscopy (SEM) images revealed asymmetric hollow tadpole-like nanostructures composed of AuNRs and an open hollow silica tail (Fig. 2a–c). Elemental mapping showed that all expected elements, including silicon, oxygen (from SiO_2_), and gold (from AuNR), could be detected and matched well with the relative positions in the nanotadpoles (Fig. 2d). Nanotadpoles had an average body length of 271.13 ± 19.32 nm and a hollow tail with a dedicated opening of 78.73 ± 11.88 nm at the end of the hollow tail (Fig. 2e). The UV–vis-NIR spectra (Fig. 2f) showed a broad surface plasmon resonance (SPR) peak of nanotadpoles in the range 800–1000 nm. The temperature of the nanotadpole solution (concentration: 100 μg mL^−1^) increased to ~ 41, 49, and 59 °C under 980 nm laser irradiation at varied power densities of 0.5, 1.0, and 2.0 W cm^−2^ for 10 min, respectively (Fig. 2g). We further investigated the motion performance of the PCM nanotadpoles. The trajectories of randomly selected nanorobots (n = 5) were tracked from the recorded videos using ImageJ under NIR irradiation with varied power densities, and the corresponding mean square displacement (MSD) was calculated (Fig. 2h, i). The average MSD of the nanorobot trajectories increased with time. The effective diffusion coefficient (*D*) was then calculated. The diffusion coefficient of the nanotadpole nanorobots increased from the Brownian diffusion value (~ 3.15 μm^2^ s^−1^) in the absence of NIR laser to 5.53 μm^2^ s^−1^ at 1.0 W cm^−2^ laser power, and 10.80 μm^2^ s^−1^ at 2.0 W cm^−2^ laser power (Fig. 2j). The motion is due to a thermophoretic mechanism.

### Preparation and Characterization of RSF Nanofibril Solutions

**Fig. 3:**
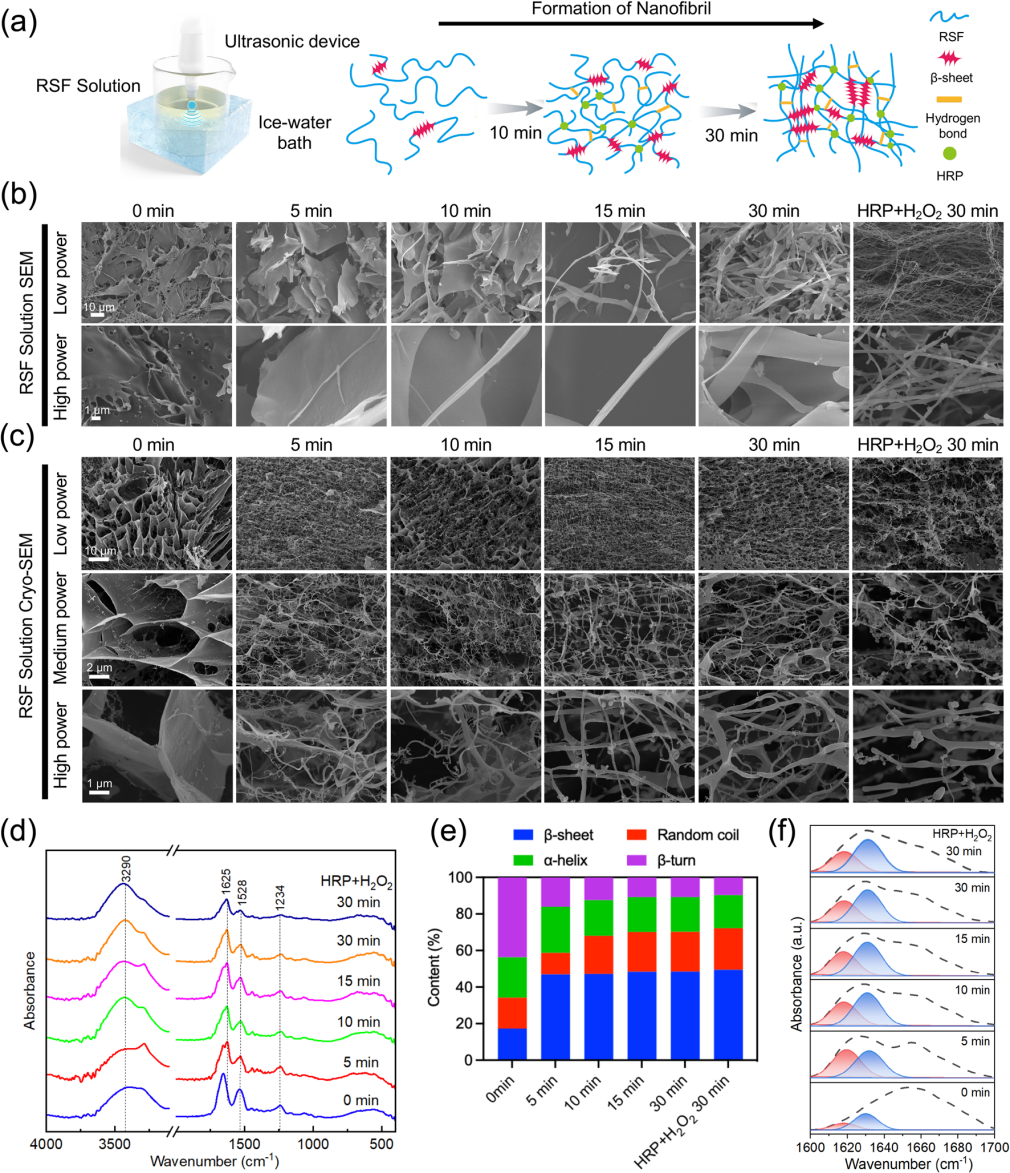
Characterization of RSF nanofibril solutions. **a** Schematic of the rapid preparation of RSF nanofibril solution by ultrasonication. **b** SEM images of RSF nanofibril solutions at different sonication times. **c** Cryo-SEM images of RSF nanofibril solutions at different sonication times. **d** FTIR spectra of RSF nanofibril solutions at different sonication times. **e** Secondary structure content of RSF nanofibril solutions at different sonication times. **f** Fitted single peaks of β-sheet content of RSF nanofibril solutions in the range of 1600–1700 cm^−1^ at different sonication times

Self-assembly of RSF nanofibrils can only be induced under stringent conditions, such as with organic solvents, at high temperatures and pH values, it involves the slow growth of nanofibrils over a period of several days [32–34]. Sonication is a highly efficient and controlled technique for RSF hydrogel formation. During sonication, mechanical vibration causes bubble formation and collapse, which increases the formation of β-sheet [23]. The pre-enzymatically crosslinked RSF solution was subjected to nanofibrillation by sonication for 30 min (300 W), which is faster and easier than the previously reported natural assembly of RSF nanofibrils with ethanol (Fig. 3a) [32]. SEM was used to observe the morphological changes of the RSF solution after sonication. As the sonication time increased from 0 to 30 min, the RSF nanofibrils became denser and more homogeneous, whereas only a small number of RSF nanofibrils were formed under short sonication conditions, and most of the RSF showed a laminar structure (Fig. 3b). However, the formation of RSF nanofibrils containing horseradish peroxidase (HRP) and H_2_O_2_ was more evident than those without HRP and H_2_O_2_ during sonication, confirming that the enzyme crosslinker promoted the formation of RSF nanofibrils (Figs. 3b and S2) with sonication times of 5, 10, and 15 min). In this work, HRP + H_2_O_2_ was used to generate di-tyrosine pre-crosslinking of RSF chains which induced the interaction between RSF chains and shortened spatial distances of molecule chains in solutions. During sonication of pre-crosslinked RSF solutions, the RSF molecule chains pretreated with HRP enzyme crosslinking have a greater chance and more efficient on forming nanofibrils with uniform β-sheet nanocrystal structure in 30 min. The morphology of RSF nanofibrils was observed using cryo-SEM (Fig. 3c). The sonicated RSF solutions exhibited a more pronounced morphology than those not sonicated, indicating that sonication may have accelerated the formation of RSF nanofibril structures. However, regardless of the duration of sonication, all treated RSF samples showed a significant increase in the number of nanofibrils, forming a network-like structure.

The structural variations of the RSF solutions were further analyzed using FTIR, the FTIR spectra of the sonicated RSF solution (absorption peak at 1234, 1528, and 1625 cm^−1^) demonstrated that β-sheet was the main conformation of the RSF nanofibrils (Fig. 3d). The amide I region mainly represents the C = O stretching vibrations in the protein structure. Proteins have different secondary structures including β-sheet (1600–1640 cm^−1^), random coil (1640–1650 cm^−1^), α-helixe (1650–1660 cm^−1^), and β-turn (1660–1700 cm^−1^). The amide I FTIR spectra were subsequently subjected to curve-fitting analysis (Fig. 3e, f). Notably, all sonicated RSF solutions exhibited a substantial abundance of β-sheet, with the content increasing from 17.3% (0 min) to 48.4% (30 min) with prolonged sonication time. Remarkably, the presence or absence of HRP and H_2_O_2_ did not induce any significant alteration in the β-sheet content (49.4%). XRD studies of the crystallinity of RSF nanofibrils further confirmed the FTIR results (Fig. S3). After sonication for 30 min, the RSF nanofibril solutions, with or without HRP and H_2_O_2_ showed a distinct XRD peak at approximately 20.29°, which was characteristic of the β-sheet structure. Meanwhile, the peaks of RSF nanofibril solutions containing HRP and H_2_O_2_ were sharper, indicating that the β-sheet crystals were more organized. These quantitative findings unequivocally demonstrate that ultrasonically prepared RSF nanofibrils facilitate a structural transition from random coil/α-helix conformation to β-sheet conformation while concurrently enhancing hydrogen bonding interactions with increasing sonication time. This RSF nanofibril formation may be attributed to the alteration in the hydration status of hydrophobic moieties within protein chains, resulting in the accelerated formation of hydrophobic physical cross-links such as intra- and interchain interactions associated with β-sheet. Similar effects induced by sonication have been reported in the literature concerning the expedited self-assembly, aggregation, and gelation processes observed in polymers due to chain breaks and the synthesis of self-assembled peptides [24]. Moreover, the incorporation of HRP enzyme crosslinking into RSF chain segments along with increased intermolecular β-sheet content enables a more efficient formation and orderly arrangement of RSF nanofibrils.

### Preparation and Characterization of Nanorobots/Thr/RSF Nanofibril Hydrogels

**Fig. 4:**
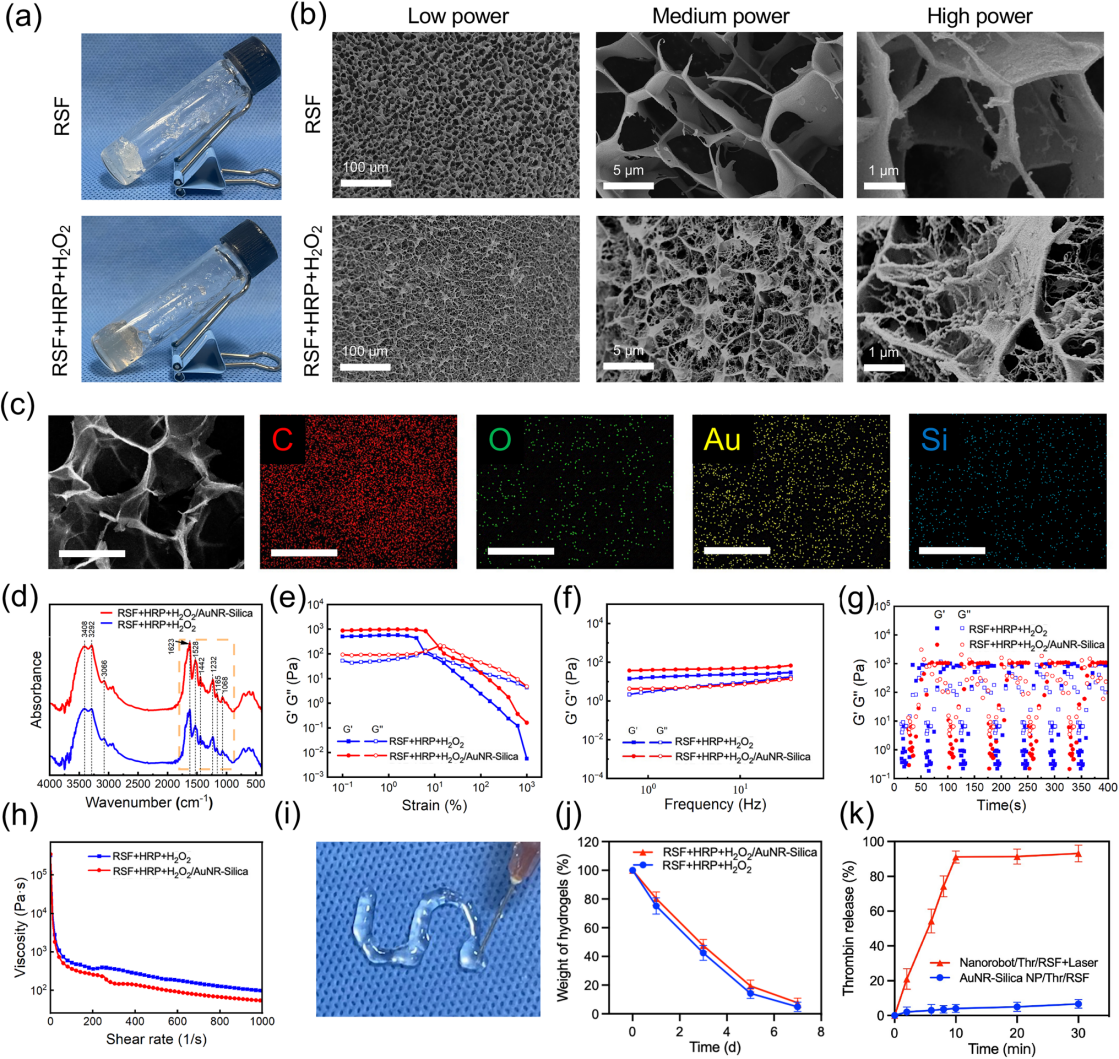
Characterization and mechanical properties of injectable RSF and Nanorobot/RSF nanofibril hydrogels. **a** Image of RSF (with or without HRP and H_2_O_2_) nanofibril hydrogel. **b** Cryo-SEM image of RSF (with or without HRP and H_2_O_2_) nanofibril hydrogel. **c** SEM images of C, O, Au, and Si of AuNR-Silica NP/RSF nanofibril hydrogels. Scale bar, 20 μm. **d** FTIR spectra of RSF (with or without HRP and H_2_O_2_) nanofibril hydrogel. **e–h** Strain scans, frequency scans, shear recovery tests, and viscosity measurements of RSF and AuNR-Silica NP/RSF nanofibril hydrogels. **i** The “S” shape of RSF nanofibril hydrogels after injection. **j** Degradation of RSF and AuNR-Silica NP/RSF nanofibril hydrogels in protease XIV for 7 days. Data are presented as the mean ± s.d. Statistical analysis was performed using two-tailed Student’s t test (n = 3 replicates; **P < 0.05, ***P < 0.001, ****P < 0.0001). **k** Thr release from Nanorobot/Thr/RSF nanofibril hydrogels under laser irradiation. Data are presented as the mean ± s.d. Statistical analysis was performed using two-tailed Student’s t test (n = 3 replicates; **P < 0.05, ***P < 0.001, ****P < 0.0001)

Injectable AuNR-Silica NP/Thr/RSF nanofibril hydrogels were prepared using a simple two-step sonication and ultrafiltration process. Under centrifugal conditions, RSF nanofibrils readily intertwine to form intricate 3D networks through concentration. Consequently, ultrafiltration facilitates small-scale aggregation of RSF nanofilaments, leading to the formation of robust RSF nanofibril hydrogels within the ultrafiltration tube (Fig. S4). The overall observation of the hydrogels depicted in Fig. 4a reveals that hydrogels can be formed in RSF nanofibril solutions with and without HRP enzyme crosslinking, although their appearance remains consistent, there may be differences in color (Fig. 4a). The structure of RSF nanofibril hydrogels was characterized using cryo-SEM (Fig. 4b), which showed the porous structure of RSF nanofibril hydrogels. It was also observed that the RSF nanofibril hydrogels with HRP and H_2_O_2_ contained abundant nanofibril morphology around the pores, confirming the role of HRP enzyme crosslinking in promoting the formation of nanofibrils. Therefore, we chose RSF nanofibril hydrogels containing HRP and H_2_O_2_ with superior performance and structure to load AuNR-Silica NPs.

To demonstrate the successful loading and distribution of the AuNR-Silica NPs within the RSF nanofibril hydrogels, SEM–EDS was performed to analyze the presence of C, O, Au, and Si components (Au and Si were the metallic elements particularly present in the AuNR-Silica NPs), the results confirmed the homogeneous distribution of the AuNR-Silica NPs in the RSF nanofibril hydrogels (Fig. 4c). The FTIR spectra mainly showed the enhancement of –OH stretching (3446 cm^−1^), C = O stretching (1623 cm^−1^) and C–N–H bending (1528 cm^−1^), and the peaks at 1623 and 1528 cm^−1^ showed a slight blue shift after loading with AuNR-Silica NPs, further confirming the successful incorporation of AuNR-Silica NPs in the RSF nanofibril hydrogels and the typical filamentary β-sheet conformation in the composite hydrogels (Fig. 4d).

Next, the rheological properties of the RSF and AuNR-Silica NP/RSF nanofibril hydrogels were determined. First, strain scanning experiments showed that the RSF and AuNR-Silica NP/RSF nanofibril hydrogel points were around 10%, and G’ and G” decreased with increasing strain, indicating that RSF and AuNR-Silica NP/RSF nanofibril hydrogels have shear thinning properties (Fig. 4e). Next, as shown in Fig. 4f, dynamic frequency scanning results showed that G’ was always larger than G” at progressively enhanced frequencies from 0.1 to 10 Hz, indicating the predominantly elastic behavior of RSF and AuNR-Silica NP/RSF nanofibril hydrogels (Fig. 4f). The recovery rate and ratio are important parameters for assessing the injectable properties of hydrogels. Remarkably, RSF and AuNR-Silica NP/RSF nanofibril hydrogels sustained huge strain treatment (5000%), and G’ was able to recover to 90% even after consecutive repetitions (Fig. 4g). In addition, the viscosity of the RSF nanofibril hydrogels was investigated (Fig. 4h). We found that the viscosities of the RSF and AuNR-Silica NP/RSF nanofibril hydrogels decreased with increasing shear rate. Finally, the feasibility of the rheological data was verified by syringe manipulation. As shown in Fig. 4i, the RSF nanofibril hydrogels could be injected continuously into the petri dishes with a syringe, and the formed "S" scripts were maintained in the hydrogel state, confirming the injectability of the nanofibril hydrogels. Therefore, the rheological properties of the RSF and AuNR-Silica NP/RSF nanofibril hydrogels in this study were superior to those of previously synthesized RSF nanofibril hydrogels [32, 35].

The degradation characteristics of the RSF nanofibril hydrogels were also investigated. The results showed that enzyme treatment led to significant degradation of the RSF nanofibril hydrogels and AuNR-Silica NP/RSF nanofibril hydrogels, with only a small number of components remaining after 7 days, indicating that these RSF nanofibril hydrogels could be almost completely degraded. This further confirms the suitability of RSF nanofibril hydrogels for nanorobot delivery and superior bio-applications (Fig. 4j). To further investigate the photothermal conversion ability of the Nanorobot/RSF nanofibril hydrogels, we recorded the temperature change of the sample under a 980 nm laser (2.0 W cm^−2^) for 600 s (Fig. S5 and S6). The data showed that the photothermal conversion efficiency of the Nanorobot/RSF nanofibril hydrogels could reach 66.56%. The significant photothermal effect generated by the Nanorobot/Thr/RSF nanofibril hydrogels can trigger the melting of PCM, which enables the rapid release of Thr from the nanorobots. Therefore, we investigated the release of Thr from the Nanorobot/Thr/RSF nanofibril hydrogels (AuNR-Silica NP/Thr/RSF with Laser named Nanorobot/Thr/RSF + Laser, AuNR-Silica NP/Thr/RSF without Laser named AuNR-Silica NP/Thr/RSF, both groups contained PCM). Under laser irradiation, a rapid release of Thr (release rate up to approximately 90%) was observed. In the absence of laser irradiation, Thr release was almost absent, indicating that the Thr release was dependent on the PCM (Fig. 4k). The photothermally triggered release of Thr enabled the safe properties of the Nanorobot/Thr/RSF nanofibril hydrogels without leakage of the loaded drugs.

### Biocompatibility of Nanorobot/Thr/RSF Nanofibril Hydrogels

**Fig. 5:**
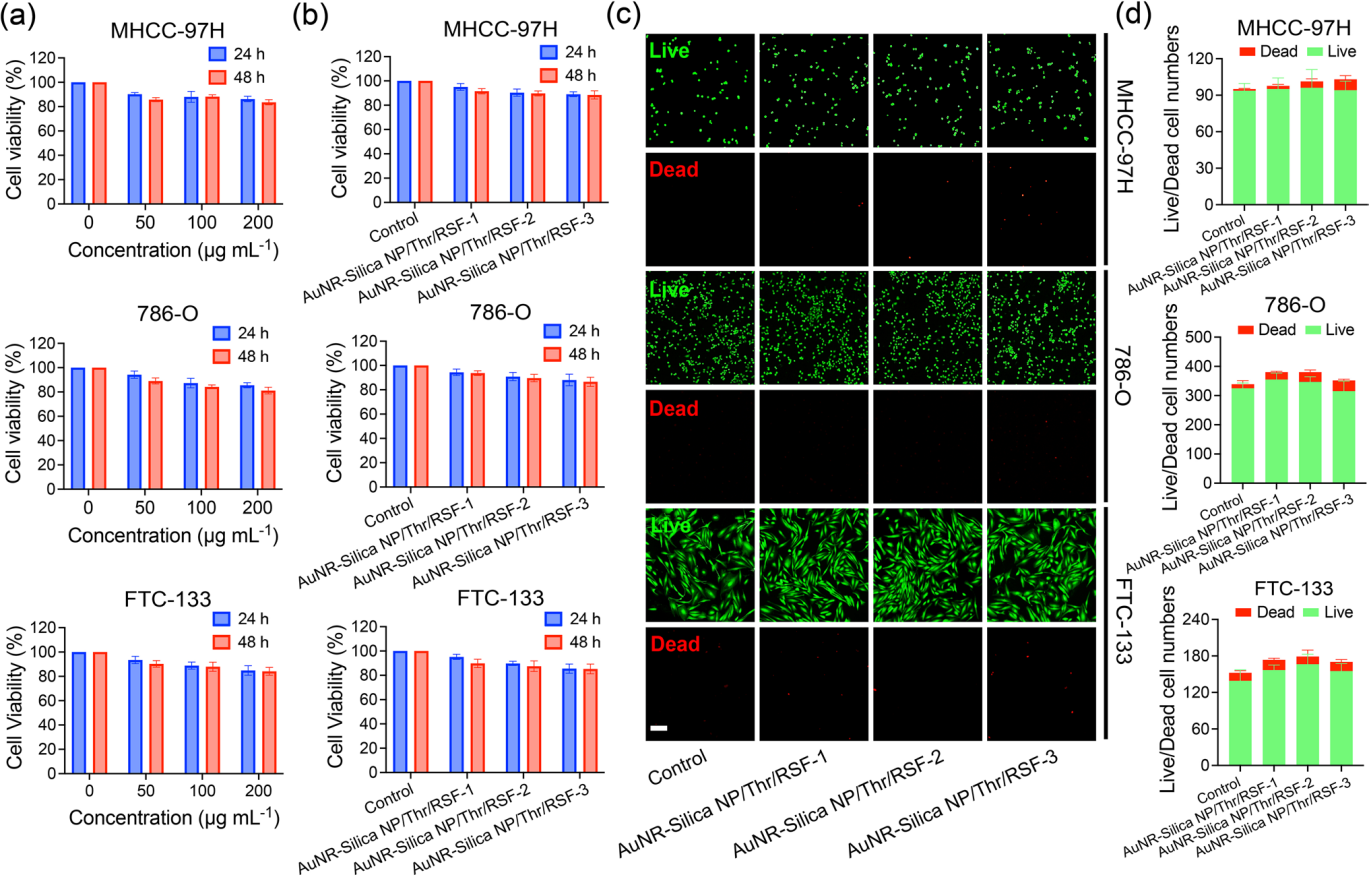
In vitro biocompatibility of AuNR-Silica NP/Thr/RSF nanofibril injectable hydrogels. **a** CCK8 assay of tumor cells (MHCC-97H, 786-O, and FTC-133) co-cultured with AuNR-Silica NP/Thr. Data are presented as the mean ± s.d. **b** CCK8 assay of tumor cells (MHCC-97H, 786-O, and FTC-133) co-cultured with AuNR-Silica NP/Thr/RSF nanofibril hydrogels (AuNR-Silica NP/Thr/RSF-1 represents AuNR-Silica NP/Thr concentration of 50 μg mL^−1^, AuNR-Silica NP/Thr/RSF-2 represents AuNR-Silica NP/Thr concentration of 100 μg mL^−1^, and AuNR-Silica NP/Thr/RSF-3 represents AuNR-Silica NP/Thr concentration of 200 μg mL^−1^). **c** Live/dead staining of tumor cells (MHCC-97H, 786-O, and FTC-133) treated with RSF nanofibril hydrogel and AuNR-Silica NP/Thr/RSF nanofibril hydrogel (green fluorescence for live cells and red fluorescence for dead cells. AuNR-Silica NP/Thr/RSF-1 represents AuNR-Silica NP/Thr concentration of 50 μg mL^−1^, AuNR-Silica NP/Thr/RSF-2 represents AuNR-Silica NP/Thr concentration of 100 μg mL^−1^, and AuNR-Silica NP/Thr/RSF-3 represents AuNR-Silica NP/Thr concentration of 200 μg mL^−1^). Scale bar, 200 μm. **d** Cell counting analysis of live/dead staining. Data are presented as the mean ± s.d. Statistical analysis was performed using a two-tailed Student’s t test (n = 3 replicates)

Regarding the translational value of any biomaterial in the clinic, the main concern is its biocompatibility. First, the biocompatibilities of different concentrations of AuNR-Silica NP/Thr and AuNR-Silica NP/Thr/RSF nanofibril hydrogels were examined using CCK-8 experiments. As shown in Fig. 5a, b, the CCK-8 assay showed that the tumor cell viabilities (MHCC-97H; HCC, hypervascularity in spinal metastasis) of the AuNR-Silica NP/Thr and AuNR-Silica NP/Thr/RSF nanofibril hydrogels at 24 and 48 h were similar to those of the controls. We obtained similar results in two other tumor cell lines (786-O, FTC-133; kidney and thyroid cancer, hypervascularity in spinal metastasis) and in AML-12 normal cell (Figs. S7,  S8). In addition, we performed a live/dead cell staining analysis, the tumor cells co-cultured with the AuNR-Silica NP/Thr/RSF nanofibril hydrogels maintained high biological activity (Figs. 5c, d and S9). In addition, we examined the effect of Nanorobot/Thr/RSF nanofibril hydrogels on DNA damage by fluorescent staining with γ-H2AX, the most sensitive molecule for sensing DNA damage in tumor cells [36]. The results showed a significant increase in DNA damage (red fluorescence) after photothermal therapy in MHCC-97H cells treated with Nanorobot/RSF + Laser and Nanorobot/Thr/RSF + Laser (Fig. S10). Untreated and RSF-treated cells did not exhibit red fluorescence, indicating no DNA damage. Red fluorescence was also not observed in AuNR-Silica NP/RSF nanofibril hydrogels and AuNR-Silica NP/Thr/RSF nanofibril hydrogels in the absence of laser irradiation, indicating that the AuNR-Silica NP/Thr/RSF nanofibril hydrogels did not cause DNA damage in the absence of laser irradiation. Taken together, these results confirmed the biocompatibility of the Nanorobot/Thr/RSF nanofibril hydrogels.

### In Vivo Hemostasis and Intraoperative Bleeding from HCC Spinal Metastasis

**Fig. 6:**
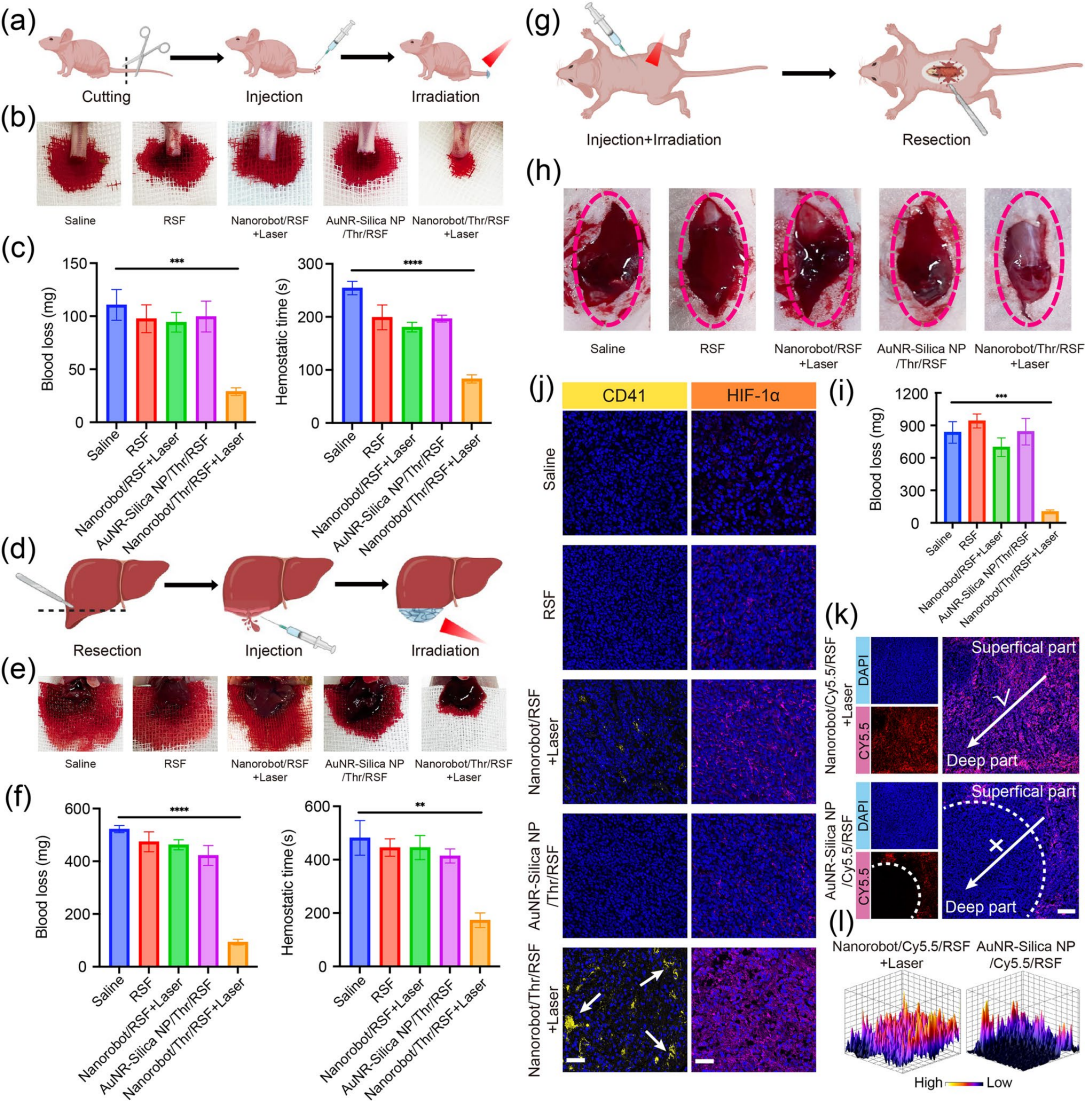
In vivo hemostasis and intraoperative bleeding from HCC spinal metastasis with Nanorobot/Thr/RSF nanofibril hydrogels. **a** Schematic diagram of the nude mice tail truncation model. **b** Photographs of tail hemorrhage in different treatment groups. **c** Blood loss and hemostasis time of different treatment groups after trans-tail truncation. Data are presented as the mean ± s.d. Statistical analysis was performed using two-tailed Student’s t test (n = 3 replicates; **P < 0.05, ***P < 0.001, ****P < 0.0001). **d** Schematic diagram of the nude mice liver resection model. **e** Photographs of hemorrhage in different treatment groups after liver resection. **f** Blood loss and hemostasis time of different treatment groups after trans-hepatic resection. Data are presented as the mean ± s.d. Statistical analysis was performed using two-tailed Student’s t test (n = 3 replicates; **P < 0.05, ***P < 0.001, ****P < 0.0001). **g** Schematic diagram of the HCC spinal metastasis nude mice resection model. **h** Photographs of bleeding in different treatment groups during HCC spinal metastasis surgery. **i** Blood loss in different treatment groups during HCC spinal metastasis surgery. Data are presented as the mean ± s.d. Statistical analysis was performed using two-tailed Student’s t test (n = 3 replicates; **P < 0.05, ***P < 0.001, ****P < 0.0001). **j** Immunofluorescence analysis of CD41 and HIF-1α in HCC spinal metastasis. Scale bar, 40 μm. **k** Depth of infiltration of Nanorobot/Cy5.5/RSF nanofibril hydrogels and AuNR-Silica NP/Cy5.5/RSF nanofibril hydrogels within the region of HCC spinal metastasis. Scale bar, 150 μm. **l** 3D fluorescence distribution of Nanorobot/Cy5.5/RSF nanofibril hydrogels and AuNR-Silica NP/Cy5.5/RSF nanofibril hydrogels in HCC spinal metastasis

Uncontrolled bleeding during surgical procedures for spinal metastasis poses a significant threat to human life [37]. In vivo evaluation of the hemostatic properties of Nanorobot/Thr/RSF nanofibril hydrogels was conducted in a nude mouse tail amputation model (Fig. 6a, b). Blood loss and hemostatic time were used as parameters to compare the efficacy of the different treatment groups (Fig. 6c). Treatment with AuNR-Silica NP/Thr/RSF nanofibril hydrogels, without laser irradiation, did not exhibit a significant hemostatic effect, mainly because thrombin was retained in PCM and was not released. However, under laser irradiation, the application of Nanorobot/Thr/RSF nanofibril hydrogels resulted in the rapid release of thrombin, leading to prompt hemostasis and significantly reduced bleeding compared to the other groups (AuNR-Silica NP/Thr/RSF with Laser named Nanorobot/Thr/RSF + Laser, AuNR-Silica NP/Thr/RSF without Laser named AuNR-Silica NP/Thr/RSF, both groups contained PCM). NIR imaging experiments confirmed that the photothermal effects of Nanorobot/Thr/RSF nanofibril hydrogel were triggered with 10 min of laser irradiation, resulting in a temperature of approximately 52.0 °C at the spinal sites in nude mice (Figs. S11,  S12). Furthermore, we validated the strong hemostatic properties of the Nanorobot/Thr/RSF nanofibril hydrogels in a nude mouse liver resection model (Fig. 6d–f).

Excessive intraoperative bleeding during spinal metastasis surgery can significantly affect the surgical field, increase procedural complexity and the risk of spinal cord injury, thus impeding complete tumor removal, and resulting in suboptimal surgical outcomes [7]. Although preoperative vascular embolization has been commonly employed in clinical practice, its efficacy remains unsatisfied. Therefore, we developed a nude mouse model of HCC spinal metastasis and surgically resected the spinal tumors to simulate intraoperative hemorrhage occurring 24 h after the minimally invasive percutaneous injection of Nanorobot/Thr/RSF nanofibril hydrogels (Fig. 6g). Our findings reveal that the control group exhibited uncontrollable intraoperative bleeding with blood permeation throughout the surgical field, whereas in the Nanorobot/Thr/RSF + Laser group, there was a significant reduction in the bleeding weight (approximately 100 mg) (Fig. 6h, i). Furthermore, it was postulated that Thr directly triggers intravascular coagulation, resulting in the formation of thrombus and subsequent obstruction of tumor vasculature [13]. To evaluate platelet aggregation within the vessels of HCC spinal metastasis, a CD41 antibody (a marker for platelet surface glycoproteins) was employed for immunostaining (Fig. 6j). The treatment of spinal tumors with Nanorobot/Thr/RSF nanofibril hydrogels combined with laser irradiation resulted in visible thrombosis within the tumor vasculature. Vascular embolism disrupts the oxygen supply and is likely to exacerbate hypoxia within the tumor microenvironment. Therefore, we further investigated hypoxia in spinal tumors using fluorescent markers specific to HIF-1α expression (Fig. 6j). Notably, the red fluorescence intensity indicated significantly stronger hypoxic regions in spinal tumors treated with Nanorobot/Thr/RSF nanofibril hydrogels with laser irradiation than in the other groups, consistent with the findings of CD41 immunofluorescence staining described above. These results suggest that reduced intraoperative bleeding during surgery for HCC spinal metastasis can be attributed to thrombus formation within tumor blood vessels triggered by Thr release, thereby effectively achieving preoperative embolization of HCC spinal metastasis by blocking the blood supply and nutrient/oxygen delivery.

To demonstrate the motility of nanorobots in RSF nanofibril hydrogels for enhanced tumor penetration and improved hemostatic performance, we compared the hemostatic efficacy of RSF nanofibril hydrogels loaded with Nanorobot/Thr + Laser (with motility) and AuNR-Silica/Thr NPs (without motility) in HCC spinal metastasis surgery. Our results revealed that motile nanorobots achieved significantly reduced bleeding compared to non-motile AuNR-Silica nanoparticles during spinal metastasis surgery (Fig. 6h). Furthermore, immunofluorescence staining demonstrated that while the AuNR-Silica NPs were primarily localized at the tumor tissue peripheries, failing to diffuse into deeper regions, the nanorobots effectively penetrated deep into the tumor tissues and were evenly distributed throughout the entire observation area (Fig. 6k, l). In conclusion, our findings highlight that incorporating motile nanorobots within RSF nanofibril hydrogels enables deep tumor penetration capabilities and effective targeting of distal tumor tissues, significantly contributing to hemostasis.

### Evaluation of Therapeutic Efficacy in the HCC Spinal Metastasis

**Fig. 7:**
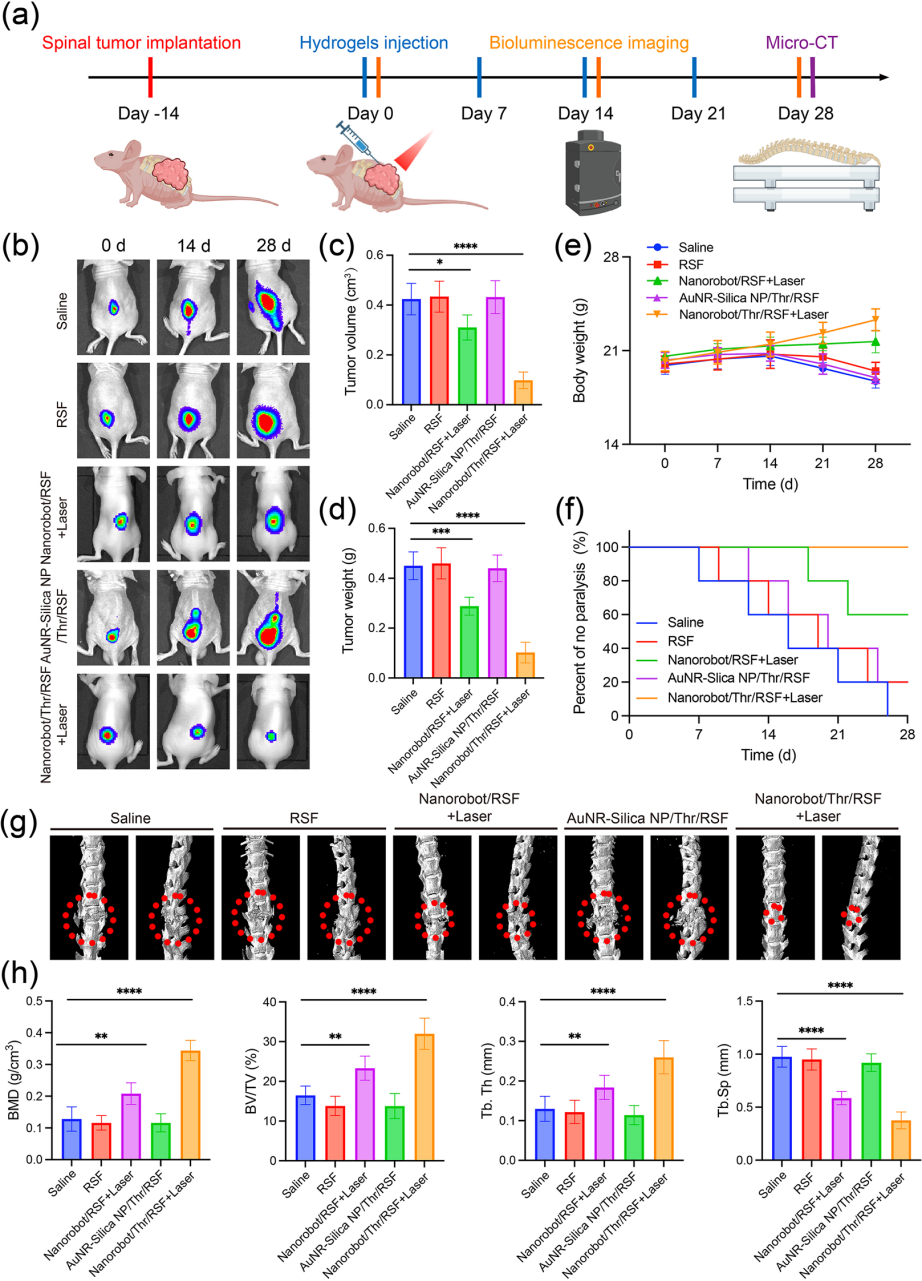
Efficacy of Nanorobot/Thr/RSF nanofibril hydrogels in the treatment of HCC spinal metastasis. **a** Timeline of different treatments in HCC spinal metastasis nude mice. **b** In vivo imaging of HCC spinal metastasis nude mice with different treatments at different time points. **c** Tumor volumes after treatment of HCC spinal metastasis from different groups. Data are presented as the mean ± s.d. Statistical analysis was performed using two-tailed Student’s t test. (n = 5 nude mice per group; **P < 0.05, ***P < 0.001, ****P < 0.0001). **d** Tumor weights after treatment of HCC spinal metastasis from different groups. Data are presented as the mean ± s.d. Statistical analysis was performed using two-tailed Student’s t test. (n = 5 nude mice per group; **P < 0.05, ***P < 0.001, ****P < 0.0001). **e** Body weights of nude mice with HCC spinal metastasis during treatment in different groups. Data are presented as the mean ± s.d. (n = 5 nude mice per group). **f** Paralysis rate in nude mice with HCC spinal metastasis during treatment in different groups. Data are presented as the mean ± s.d. (n = 5 nude mice per group). **g** Micro-CT analysis of vertebral bone destruction in each treatment group. **h** Micro-CT parameters analysis (BMD, BV/TV, Tb.Th, and Tb.Sp) of spinal tumor regions in each treatment group. Data are presented as the mean ± s.d. Statistical analysis was performed using two-tailed Student’s t test. (n = 5 nude mice per group; **P < 0.05, ***P < 0.001, ****P < 0.0001)

We further assessed the therapeutic efficacy of starvation embolization using Nanorobot/Thr/RSF nanofibril hydrogels in vivo by establishing a nude mouse model of HCC spinal metastasis. Pre-cultured human HCC cells (MHCC-97H-Luc) were inoculated into the spines of nude mice, and after 14 days, the mice were randomly divided into five groups and treated with Saline, RSF, Nanorobot/RSF + Laser (980 nm, 2.0 W cm^−2^ for 10 min), AuNR-Silica NP/Thr/RSF, or Nanorobot/Thr/RSF + Laser (980 nm, 2.0 W cm^−2^ for 10 min). Bioluminescence imaging, tumor measurements, body weight monitoring, tissue immunofluorescence, and micro CT were performed at different time points to evaluate the treatment outcomes (Fig. 7a). Bioluminescence imaging and specimen mapping results demonstrated that the Nanorobot/Thr/RSF + Laser treatment group exhibited significant inhibition of tumor growth compared to the saline group (Figs. 7b and S13). Under laser irradiation, the Nanorobot/Thr/RSF nanofibril hydrogel induced vascular occlusion, impeding tumor growth through nutrient and blood supply deprivation. It also significantly reduced HCC spinal metastasis in nude mice by decreasing both the tumor volume and weight (Fig. 7c, d). The Nanorobot/Thr/RSF + Laser group displayed smaller tumors in the spine than the RSF and AuNR-Silica NP/Thr/RSF groups, indicating that the photothermal effects partially contributed to the inhibition of tumor growth. Complete embolization of nutrient-providing blood vessels resulted in necrosis of a substantial portion of the tumor, as confirmed by TUNEL experiments Figs. S14,  S15). In addition, no significant weight loss was observed in the Nanorobot/RSF + Laser and the Nanorobot/Thr/RSF + Laser groups (Fig. 7e). Therefore, starvation embolization therapy mediated by Nanorobot/Thr/RSF nanofibril hydrogels is more suitable for treating HCC spinal metastasis.

Once the tumor has metastasized to the spine, it tends to exert pressure on the spinal cord, leading to paralysis of the lower limbs [38]. Consequently, lower limb paralysis represents a significant complication associated with HCC spinal metastasis. The incidence of paralysis was the highest in the saline, RSF, and AuNR-Silica NP/Thr/RSF groups. However, a decrease in the incidence of paralysis was observed in the Nanorobot/RSF + Laser group. Importantly, no risk of paralysis was observed in the Nanorobot/Thr/RSF + Laser group (Fig. 7f). Bone destruction in the spinal vertebrae can indirectly reflect tumor progression. Three-dimensional reconstructive micro-CT was used to analyze bone destruction within the tumor. The CT-reconstructed images revealed that the Nanorobot/Thr/RSF + Laser group exhibited the most potent inhibitory effect on bone destruction secondary to tumor growth, accompanied by a significant alleviation of internal osteolysis (Fig. 7g). Conversely, partial bone destruction was observed in the Nanorobot/Thr/RSF + Laser group (Fig. 7h). Furthermore, quantitative assessment of the bone microstructure using parameters such as bone mineral density (BMD), bone volume fraction (BV/TV), trabecular thickness (Tb. Th), and trabecular separation (Tb. Sp) demonstrated notable bone invasion in the Saline, RSF, and AuNR-Silica NP/Thr/RSF groups. However, the administration of photothermal therapy effectively suppressed bone invasion and destruction, particularly in the Nanorobot/Thr/RSF + Laser group. In contrast, Tb. Sp showed an opposite trend, indicating that the combined treatment with Nanorobot/Thr/RSF nanofibril hydrogels and NIR treatment effectively suppressed bone destruction. Finally, the therapeutic efficacy of the Nanorobot/Thr/RSF nanofibril hydrogels was validated by in vivo imaging in two additional nude mouse models of spinal metastasis from renal and thyroid cancers (Figs. S16,  S17). Overall, these findings highlight the excellent efficacy of Nanorobot/Thr/RSF nanofibril hydrogels against spinal metastasis.

### Inhibiting HCC Spinal Metastasis Recurrence

**Fig. 8:**
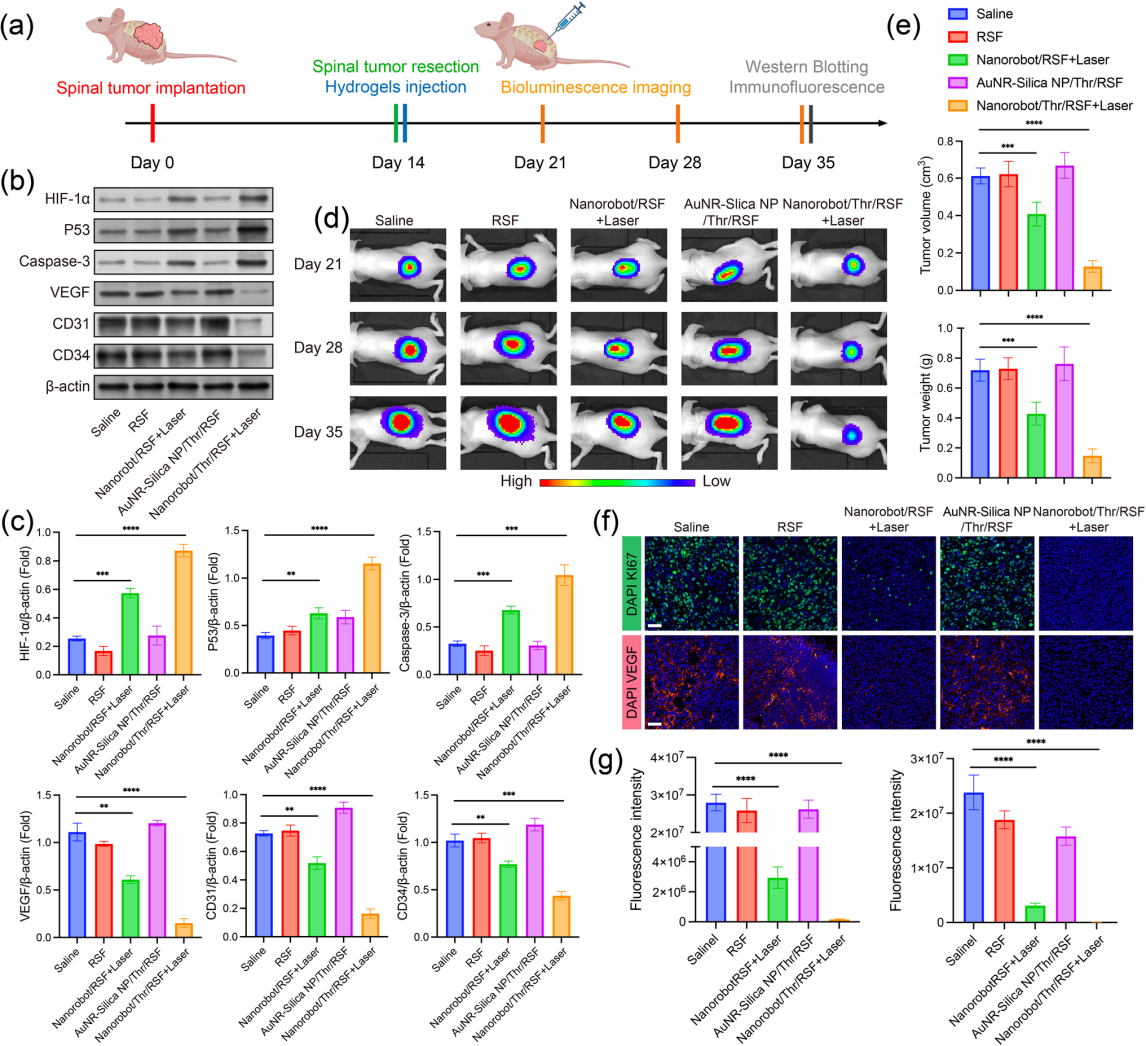
Nanorobot/Thr/RSF nanofibril hydrogels for inhibiting postoperative recurrence of HCC spinal metastasis. **a** Experimental schematic of recurrence of surgically resected HCC spinal metastasis. **b** Western blot analysis of tumor protein expression after HCC spinal metastasis surgery, including HIF-1α, P53, Caspase-3, VEGF, CD34, and CD31. **c** Quantitative analysis of protein expression, including HIF-1α, P53, Caspase-3, VEGF, CD34, and CD31. Data are presented as the mean ± s.d. Statistical analysis was performed using two-tailed Student’s t test (n = 3 replicates; **P < 0.05, ***P < 0.001, ****P < 0.0001). **d** In vivo bioluminescence imaging of MHCC-97H-Luc tumors after resection of HCC spinal metastasis. **e** Recurrent tumor volume and weight after resection of HCC spinal metastasis. Data are presented as the mean ± s.d. Statistical analysis was performed using two-tailed Student’s t test (n = 5 nude mice per group; **P < 0.05, ***P < 0.001, ****P < 0.0001). **f** Ki67 and VEGF immunofluorescence analysis of recurrent tumors after resection of HCC spinal metastasis. Scale bar, 40 μm. **g** Ki67 and VEGF fluorescence intensity of recurrent tumors after resection of HCC spinal metastasis. Data are presented as the mean ± s.d. Statistical analysis was performed using two-tailed Student’s t test (n = 3 replicates; **P < 0.05, ***P < 0.001, ****P < 0.0001)

Spinal tumors, particularly metastatic tumors, exhibit a high propensity for recurrence following surgical intervention [39]. To verify the effectiveness of Nanorobot/Thr/RSF nanofibril hydrogels in preventing spinal tumor recurrence, visible tumors were surgically resected, on the 14th day after MHCC-97H cells were inoculated into the spine of nude mice, under a small incision to establish a resection model of postoperative spinal metastasis tumors. Nanorobot/Thr/RSF nanofibril hydrogels were injected into the resected cavity (Fig. 8a).

Initially, western blot analysis was performed to investigate the potential mechanisms underlying the postoperative recurrence of spinal tumors (Fig. 8b, c). In the Nanorobot/Thr/RSF + Laser group, embolization induced by the vascular supply significantly upregulated HIF-1α protein expression, as well as P53 and Caspase-3 protein expressions. Notably, the therapeutic efficacy of the Nanorobot/RSF + Laser group was relatively modest because it only reduced VEGF secretion and prevented angiogenesis through photothermal effects without completely severing the tumor nutrient supply. Interestingly, the downregulation of CD31 and CD34 protein expression further signified that the Nanorobot/Thr/RSF + Laser group possessed the ability to induce apoptosis in tumor vascular endothelial cells, effectively disrupting blood and nutrient supply through a dual mechanism involving anti-angiogenesis and thrombosis. Consequently, this intervention exhibits potent efficacy in inhibiting the post-surgical recurrence of spinal tumors. This confirms our hypothesis that the postoperative recurrence of spinal tumors primarily relies on the blood supply and nutrition of the blood vessels.

Postoperative recurrence of spinal tumors was monitored with bioluminescent signals emitted by MHCC-97H-Luc cancer cells. Bioluminescence imaging revealed that the Nanorobot/Thr/RSF + Laser group exhibited the greatest inhibition of postoperative spinal tumor recurrence compared with the other treatment groups (Fig. 8d), confirmed further by corresponding measurements of spinal tumor volumes and weights (Fig. 8e). Ki67, a widely used proliferation marker for predicting tumor recurrence, was analyzed by immunofluorescence staining, along with VEGF expression in recurrent tumors. Significantly lower Ki67 expression was observed in the Nanorobot/Thr/RSF + Laser group than in the other groups, indicating a reduced likelihood of spinal tumor recurrence after surgery. Additionally, the fluorescent expression of VEGF, CD31, and CD34 was significantly decreased, consistent with previous findings from western blot analysis (Figs. 8f, g and S18– S21). The mechanism underlying the starvation embolization shown by the Nanorobot/Thr/RSF + Laser group involved Thr-induced thrombus formation and AuNR-mediated spread to neighboring peripheral vessels, as well as photothermal effects, leading to reduced secretion of angiogenesis-promoting chemical signals by tumor cells, thereby inhibiting tumor angiogenesis. Overall, our study demonstrated that Nanorobot/Thr/RSF + Laser treatment for HCC spinal metastasis exhibited superior efficacy in slowing postoperative recurrence and holds potential for clinical applications.

### In Vivo Biological Safety Evaluation

Although the newly developed Nanorobot/Thr/RSF nanofibril hydrogels showed promise for improving clinical efficacy, it was crucial to address potential systemic toxicity concerns. To assess in vivo biosafety of the Nanorobot/Thr/RSF nanofibril hydrogels, sections of the major organs (including the heart, liver, spleen, lungs, and kidneys) were collected from the nude mice on the final day of treatment and subjected to H&E staining. No significant inflammatory response or organ damage was observed in the major organs of the nude mice treated with Nanorobot/Thr/RSF nanofibril hydrogels with Laser compared to the other treatment groups (Fig. S22). H&E staining results demonstrated a favorable biosafety profile of the Nanorobot/Thr/RSF nanofibril hydrogels. Additionally, routine blood analyses including white blood cells (WBC), lymphocytes (Lymph#), granulocytes (Gran#), red blood cells (RBC), hemoglobin (HGB), platelets (PLT), alt alanine aminotransferase (ALT), aspartate transaminase (AST), albumin (ALB), uric acid (UA), blood urea nitrogen (BUN), and creatinine (CR) were conducted to evaluate liver function, kidney function, and nutritional status of nude mice. The results revealed no significant differences among the different treatment groups for all assessed indices, which remained within normal ranges, indicating that Nanorobot/Thr/RSF nanofibril hydrogels exhibited no toxic side effects in nude mice (Figs. S23,  S24). In conclusion, this study highlights the potential clinical translational value of the newly developed Nanorobot/Thr/RSF nanofibril hydrogels in treatment of spinal metastasis.

## Conclusion

In conclusion, we developed the injectable nanorobot-loaded RSF nanofibril hydrogels using a simple two-step sonication and ultrafiltration method, which offers advantages in terms of the production process, structure, and performance. This therapeutic platform enables the spatiotemporal interruption of nutrients and blood supply during spinal metastasis surgery. This platform also promotes apoptosis of spinal metastatic tumor cells and inhibits postoperative angiogenesis to reduce tumor recurrence. The self-motile nanorobots facilitate the crossing of strong biological barriers while ensuring safe delivery of the loaded Thr into the PCM. Subsequent NIR activation enables deep penetration of metastatic spinal tumors and controlled drug-release. This approach leads to enhanced and controlled accumulation of Thr within tumor blood vessels. This starvation embolization therapy potentially addresses three key issues: intraoperative blood loss control, inhibition of the progression of spinal metastasis, and prevention of recurrence. Our minimally invasive treatment platform introduces advanced preoperative treatment options for HCC with spinal metastasis, thus laying the foundation for reduced surgical complications and improved outcomes.

## Supplementary Information

Below is the link to the electronic supplementary material.

Supplementary file1 (DOCX 11764 KB)
