# Supplementary material for: Injectable Nanorobot-Hydrogel Superstructure for Hemostasis and Anticancer Therapy of Spinal Metastasis
Source: Nanomicro Lett. 2024 Aug 1;16:259. doi: 10.1007/s40820-024-01469-3 (PMC11291792; doi:10.1007/s40820-024-01469-3)
Supplement: Supplementary file 1 — Supplementary file1 (DOCX 11764 KB) [file 40820_2024_1469_MOESM1_ESM.docx]

Supporting Information for

**Injectable Nanorobot-****Hydrogel Superstructure for** **Hemostasis and Anticancer Therapy of Spinal Metastasis**

Qing Chen^1, #^, Miao Yan^2, 3, #^, Annan Hu^1, #^, Bing Liang^1, #^, Hongwei Lu^1^, Lei Zhou^1^, Yiqun Ma^1^, Chao Jia^1^, Dihan Su^1^, Biao Kong^2,^ *, Wei Hong^4,^ *, Libo Jiang^1, 5,^ *, and Jian Dong^1, 5, 6,^ *

^1^Department of Orthopaedic Surgery, Zhongshan Hospital, Fudan University, Shanghai 200032, P. R. China

^2^Department of Chemistry, Fudan University, Shanghai 200438, P. R. China

^3^Department of Surgery, Yong Loo Lin School of Medicine, National University of Singapore, 1E Kent Ridge Road, Singapore 119228, Singapore

^4^Department of Geriatrics and Gerontology, Huadong Hospital, Fudan University, Shanghai 200438, P. R. China

^5^State Key Laboratory of Molecular Engineering of Polymers, Fudan University, Shanghai 200438, P. R. China

^6^Department of Orthopaedic Surgery Zhongshan Hospital Wusong Branch Fudan University, Shanghai 200940, P. R. China

^#^Qing Chen, Miao Yan, Annan Hu and Bing Liang contributed equally to this work.

*Corresponding authors. E-mail: [bkong@fudan.edu.cn](mailto:bkong@fudan.edu.cn) (Biao Kong); [drivyh@126.com](mailto:drivyh@126.com) (Wei Hong); [jiang.libo@zs-hospital.sh.cn](mailto:jiang.libo@zs-hospital.sh.cn) (Libo Jiang); [dong.jian@zs-hospital.sh.cn](mailto:dong.jian@zs-hospital.sh.cn) (Jian Dong)

**Supplementary Figures**


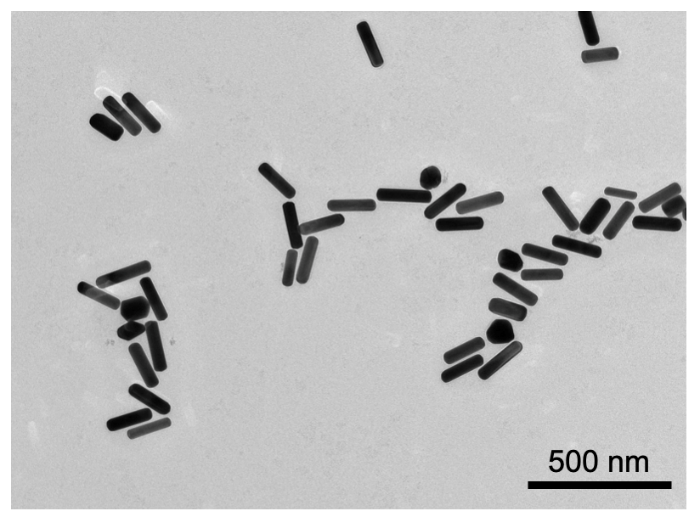


**Fig. S1** TEM image of the AuNRs


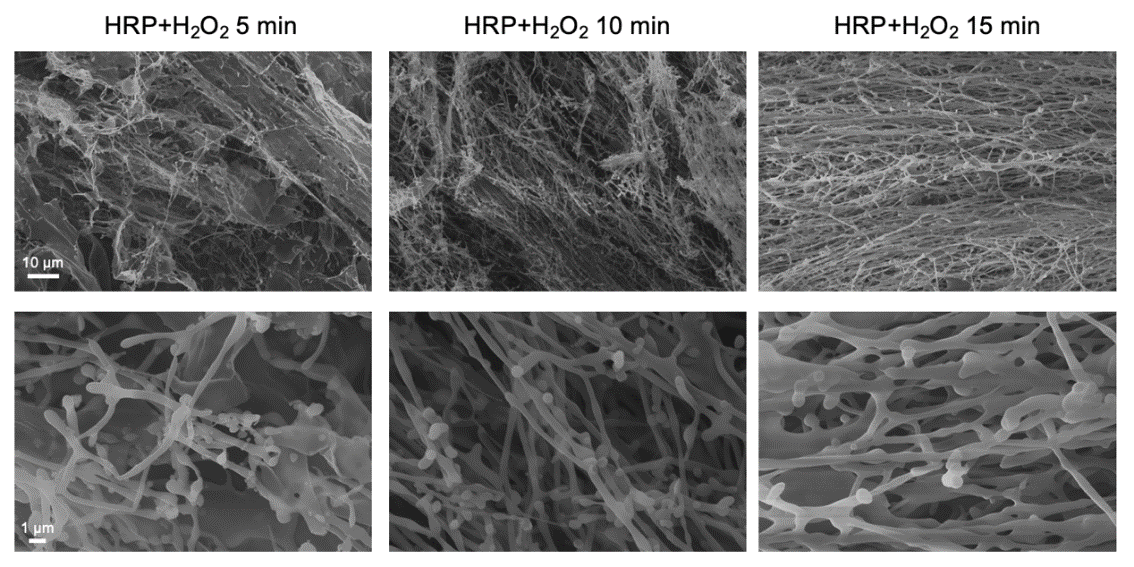


**Fig. S2** SEM images of RSF (with HRP and H_2_O_2_) nanofibril solutions at different sonication times


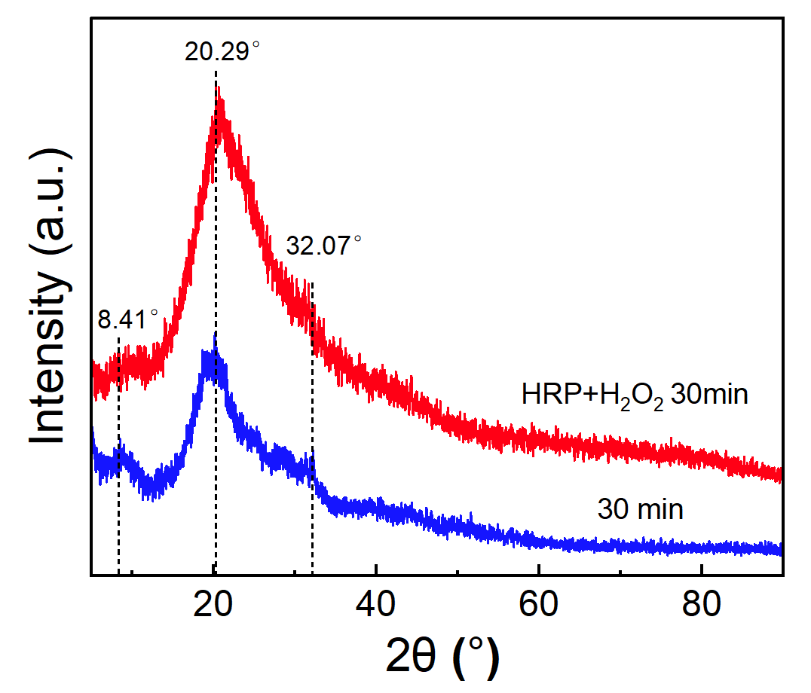


**Fig. S3** XRD analysis of RSF (with or without HRP and H_2_O_2_) nanofibril solutions after 30 min sonication

**
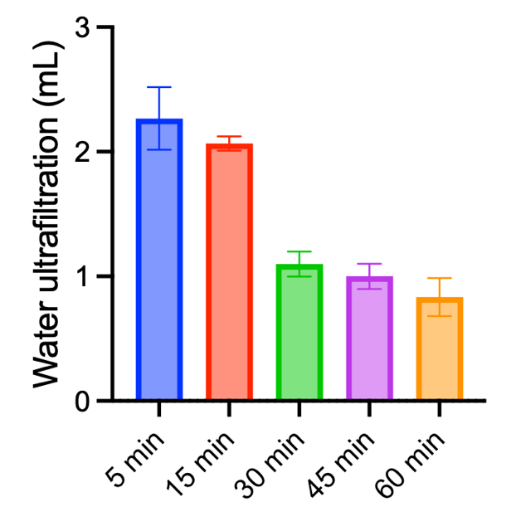
**

**Fig. S4** Water content of RSF nanofibril solutions filtered at different ultrafiltration times


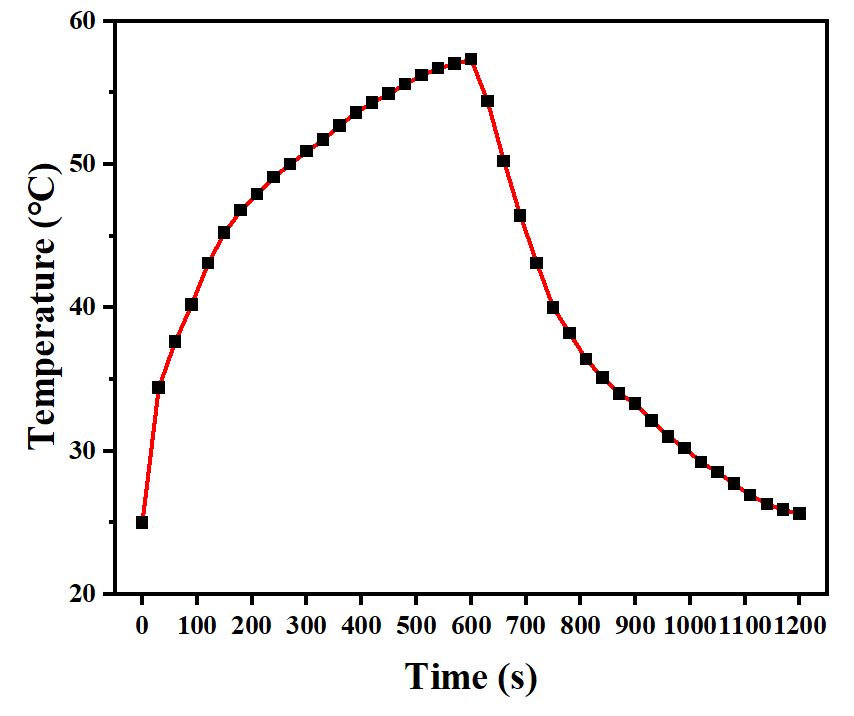


**Fig. S5** Temperature changes in Nanorobot/RSF nanofibril hydrogels cooled after 10 min of irradiation with a 980 nm laser (2.0 W cm^-2^)


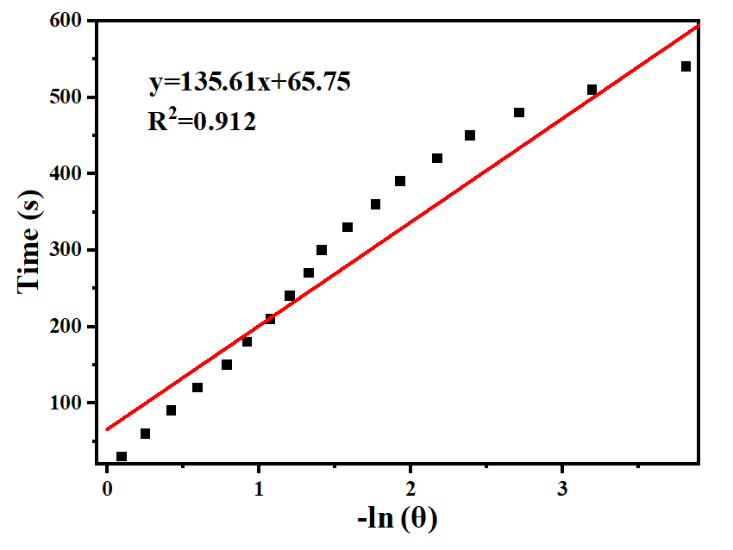


**Fig. S6** Linear time data from the cooling period versus -Ln (θ)


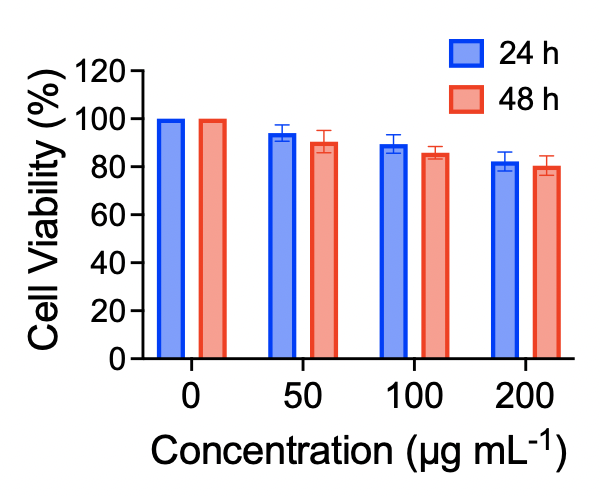


**Fig. S7** CCK8 assay of normal cells (AML-12) co-cultured with AuNR-Silica NP/Thr. Data are presented as the mean ± s.d.


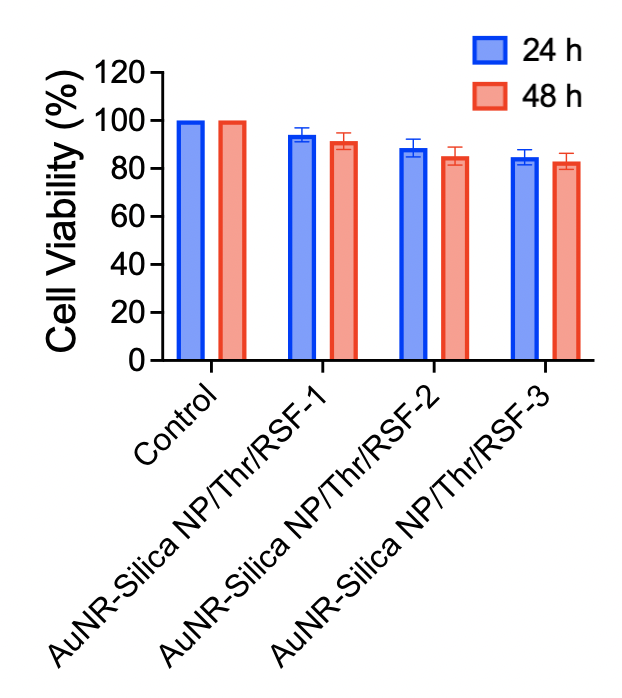


**Fig. S8** CCK8 assay of normal cells (AML-12) co-cultured with AuNR-Silica NP/Thr/RSF nanofibril hydrogels (AuNR-Silica NP/Thr/RSF-1 represents AuNR-Silica NP/Thr concentration of 50 μg/mL^-1^, AuNR-Silica NP/Thr/RSF-2 represents AuNR-Silica NP/Thr concentration of 100 μg/mL^-1^, and AuNR-Silica NP/Thr/RSF-3 represents AuNR-Silica NP/Thr concentration of 200 μg/mL^-1^)


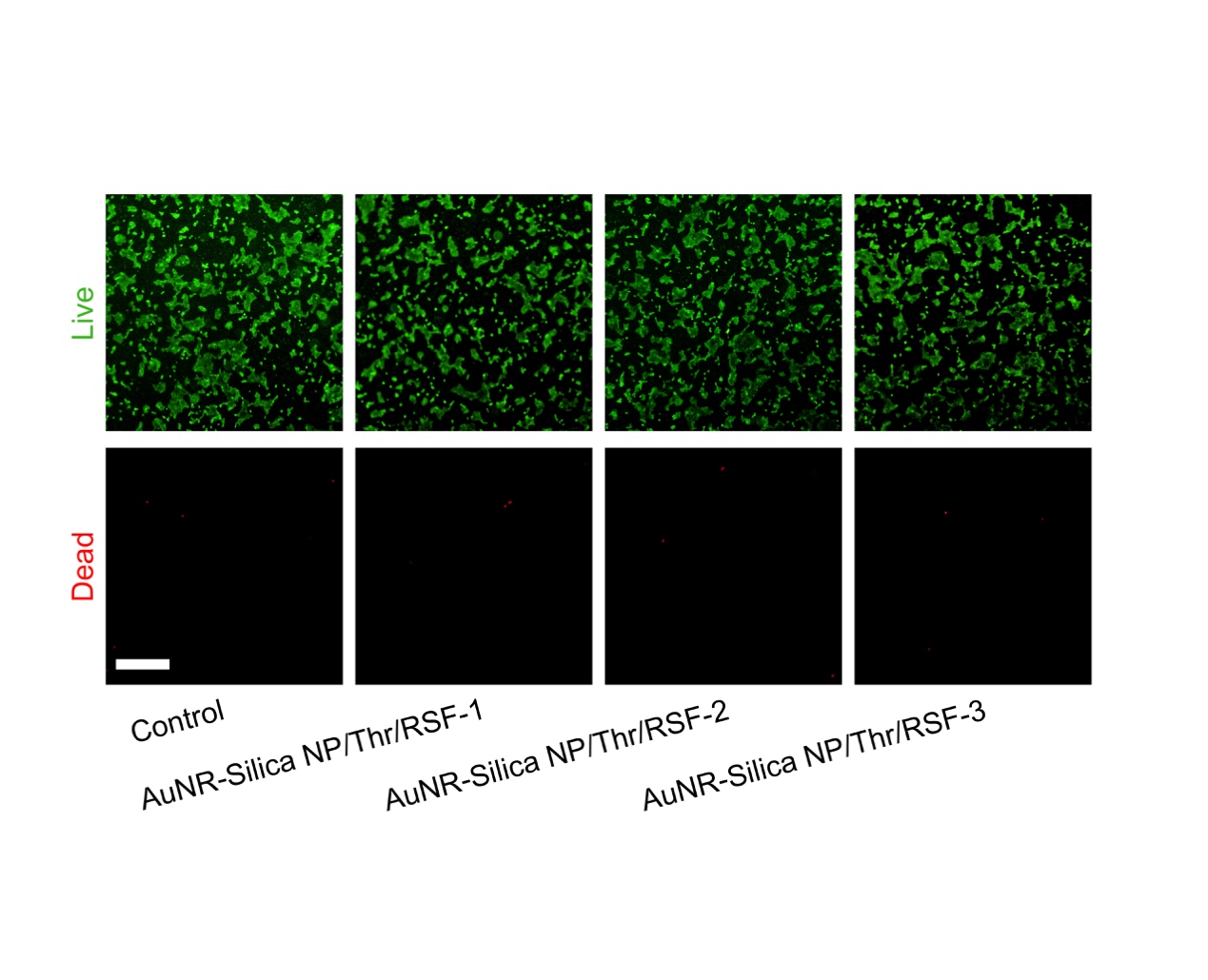


**Fig. S9** Live/dead staining of AML-12 cells treated with RSF nanofibril hydrogels and AuNR-Silica NP/Thr/RSF nanofibril hydrogels (green fluorescence for live cells and red fluorescence for dead cells. AuNR-Silica NP/Thr/RSF-1 represents AuNR-Silica NP/Thr concentration of 50 μg/mL^-1^, AuNR-Silica NP/Thr/RSF-2 represents AuNR-Silica NP/Thr concentration of 100 μg/mL^-1^, and AuNR-Silica NP/Thr/RSF-3 represents AuNR-Silica NP/Thr concentration of 200 μg/mL^-1^). Scale bar, 200 μm


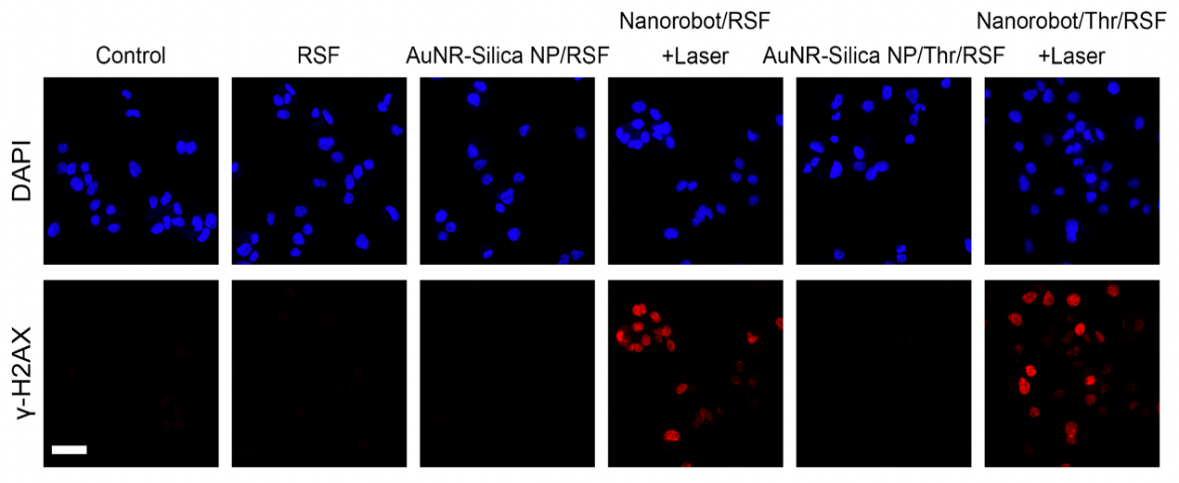


**Fig. S10** γ-H2AX fluorescence images of the MHCC-97H cells with different treatments. Scale bar, 50 μm


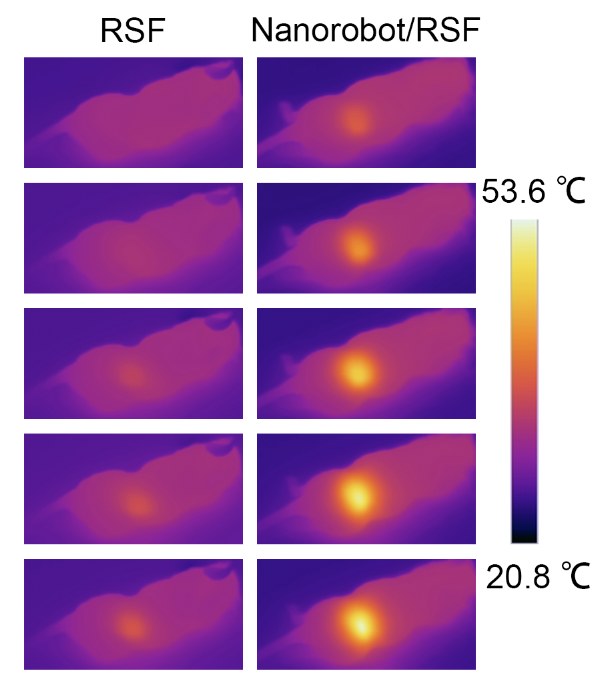


**Fig. S11** Photothermal imaging of Nanorobot/RSF nanofibril hydrogels


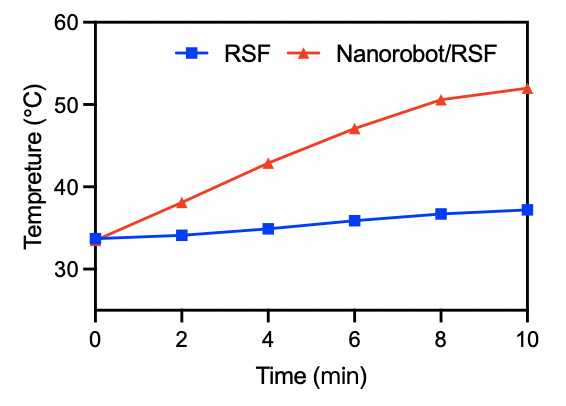


**Fig. S12** Photothermal temperature curves of Nanorobot/RSF nanofibril hydrogels


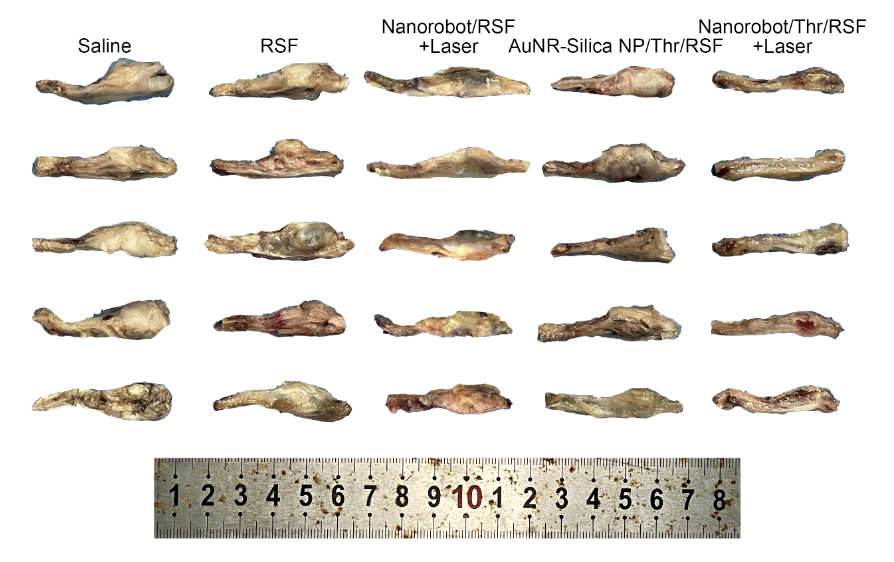


**Fig. S13** Spinal tumor specimens after different treatments of HCC spinal metastasis


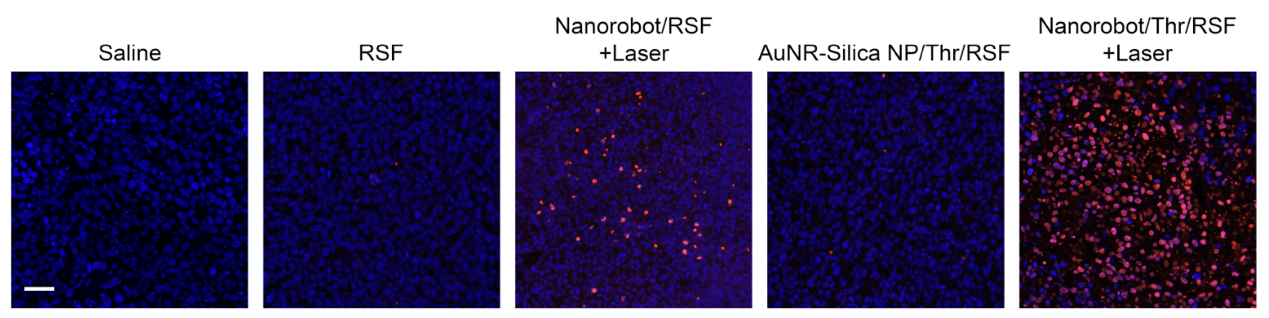


**Fig. S14** TUNEL staining after different treatments of HCC spinal metastasis. Scale bar, 40 μm


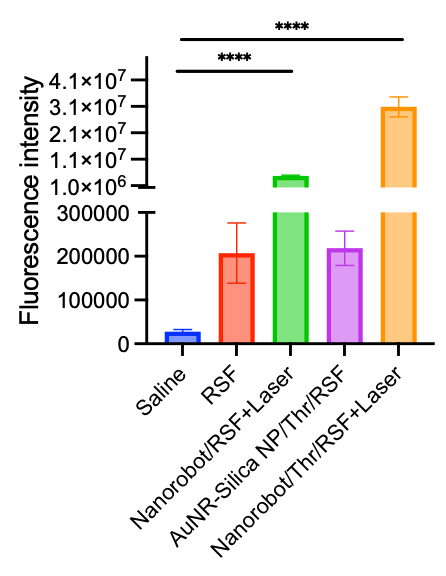


**Fig. S15** Fluorescence intensity of TUNEL staining after different treatments of HCC spinal metastasis


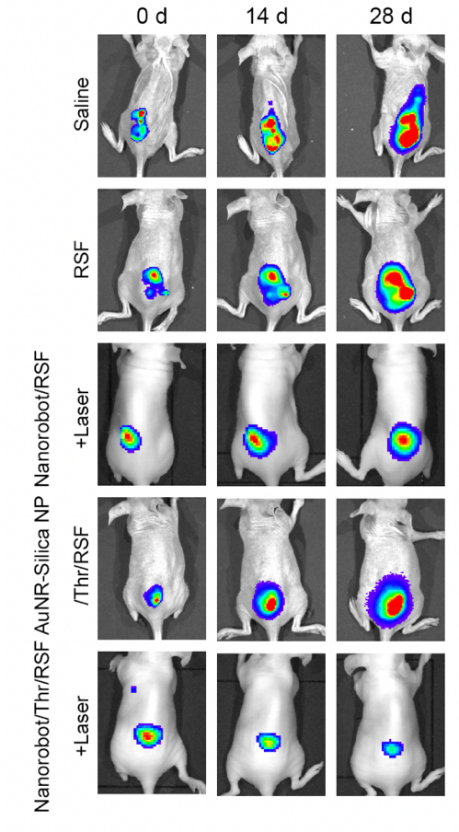


**Fig. S16** In vivo imaging of renal cancer spinal metastasis in nude mice with different treatments and at different time points


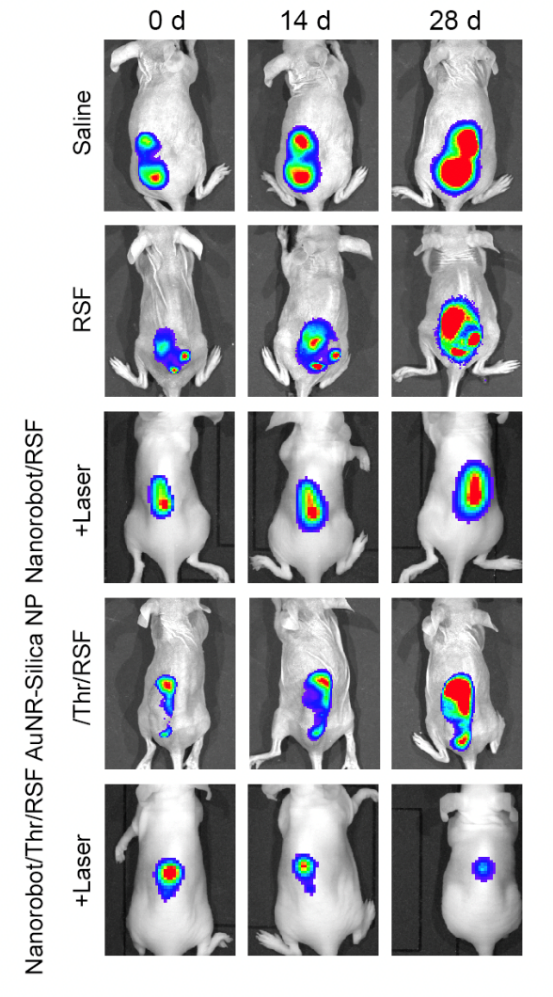


**Fig. S17** In vivo imaging of thyroid cancer spinal metastasis in nude mice with different treatments at different time points


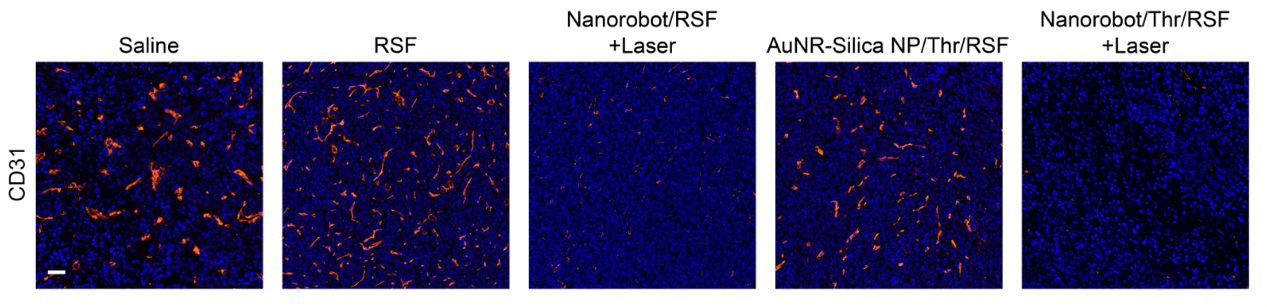


**Fig. S18** CD31 fluorescent staining in postoperative recurrence of HCC spinal metastasis. Scale bar, 50 μm

**
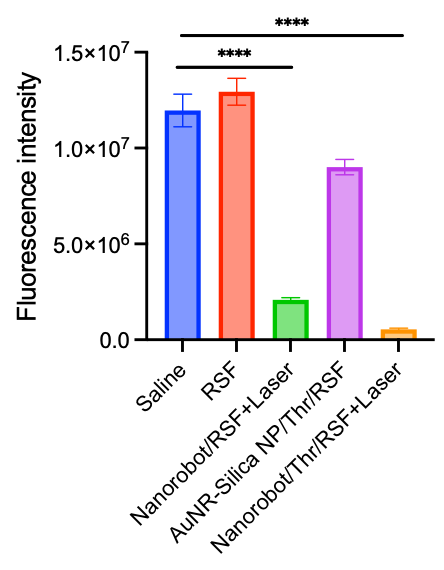
**

**Fig. S19** CD31 fluorescence intensity in postoperative recurrence of HCC spinal metastasis


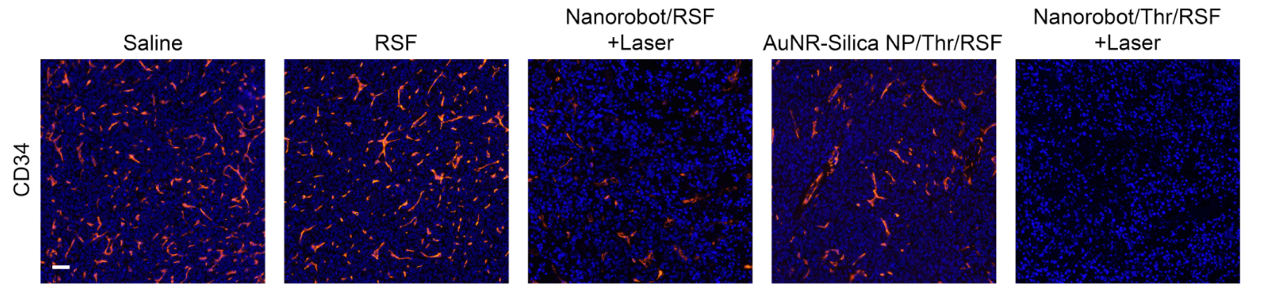


**Fig. S20** CD34 fluorescent staining in postoperative recurrence of HCC spinal metastasis. Scale bar, 50 μm


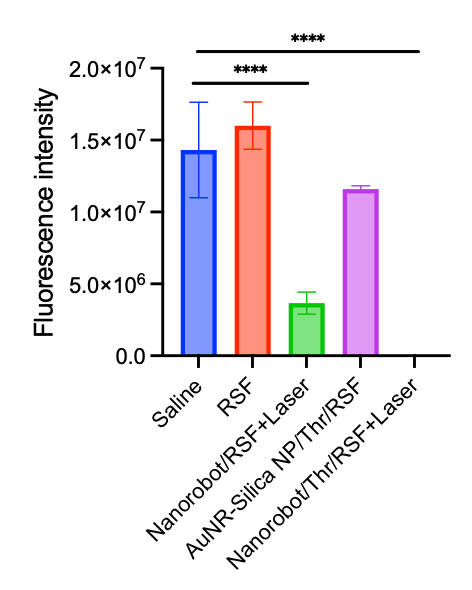


**Fig. S21** CD34 fluorescence intensity in postoperative recurrence of HCC spinal metastasis


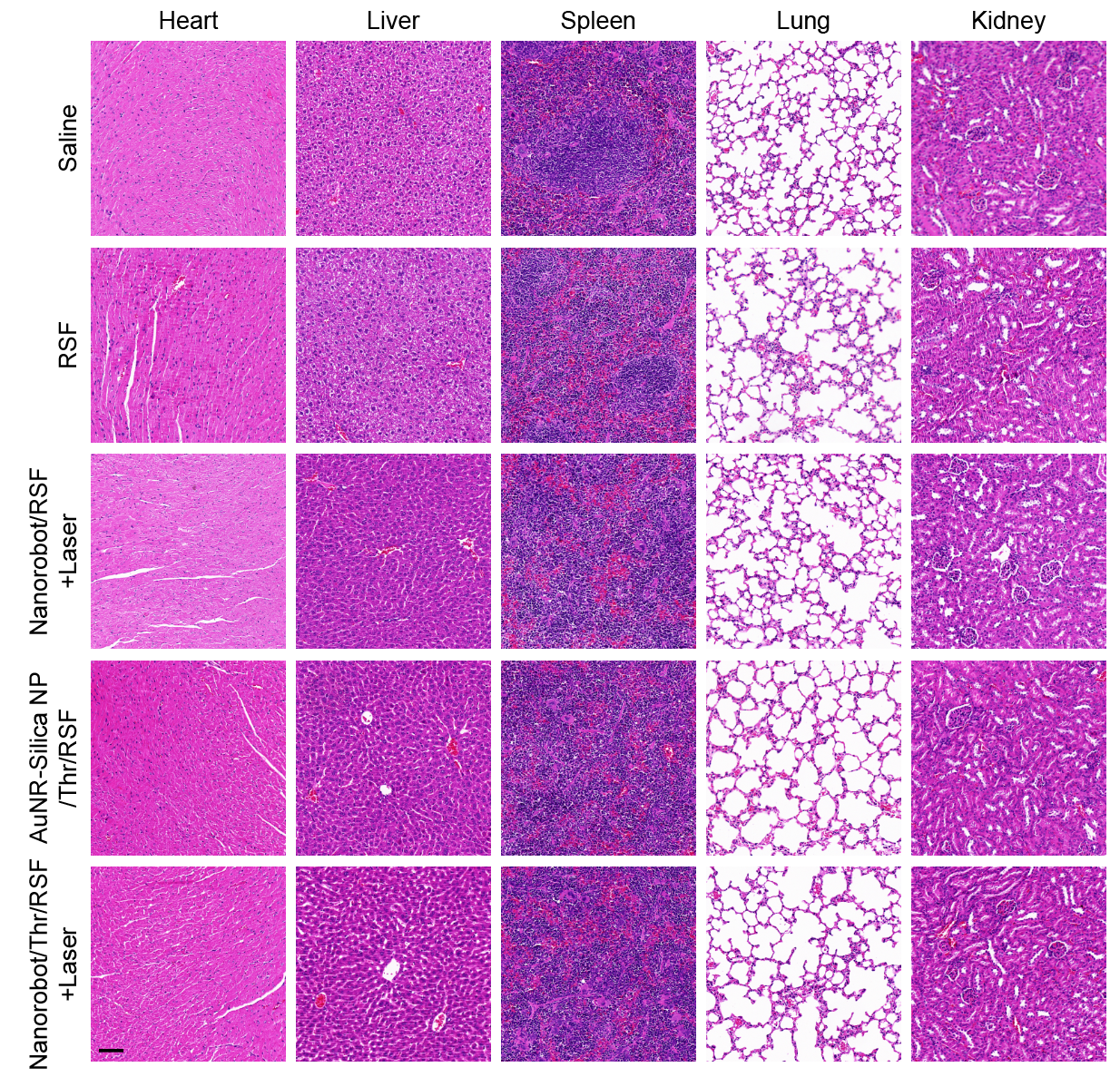


**Fig. S22** H&E staining of major organs after different treatments of HCC spinal metastasis. Scale bar, 20 μm


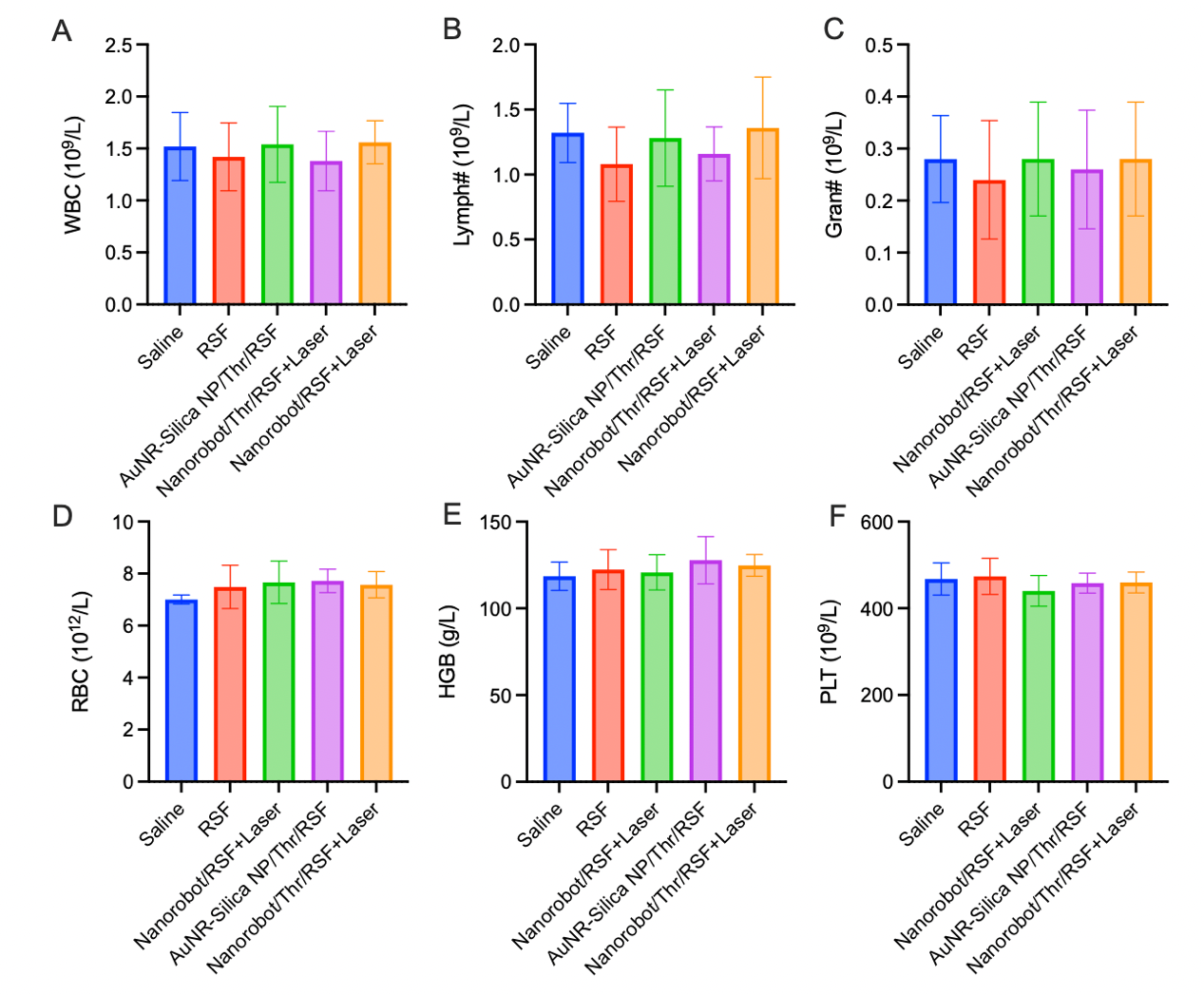


**Fig. S23** Blood counts after different treatments of HCC spinal metastasis. Blood cells (WBC), lymphocytes (Lymph#), granulocytes (Gran#), red blood cells (RBC), hemoglobin (HGB), and platelets (PLT)


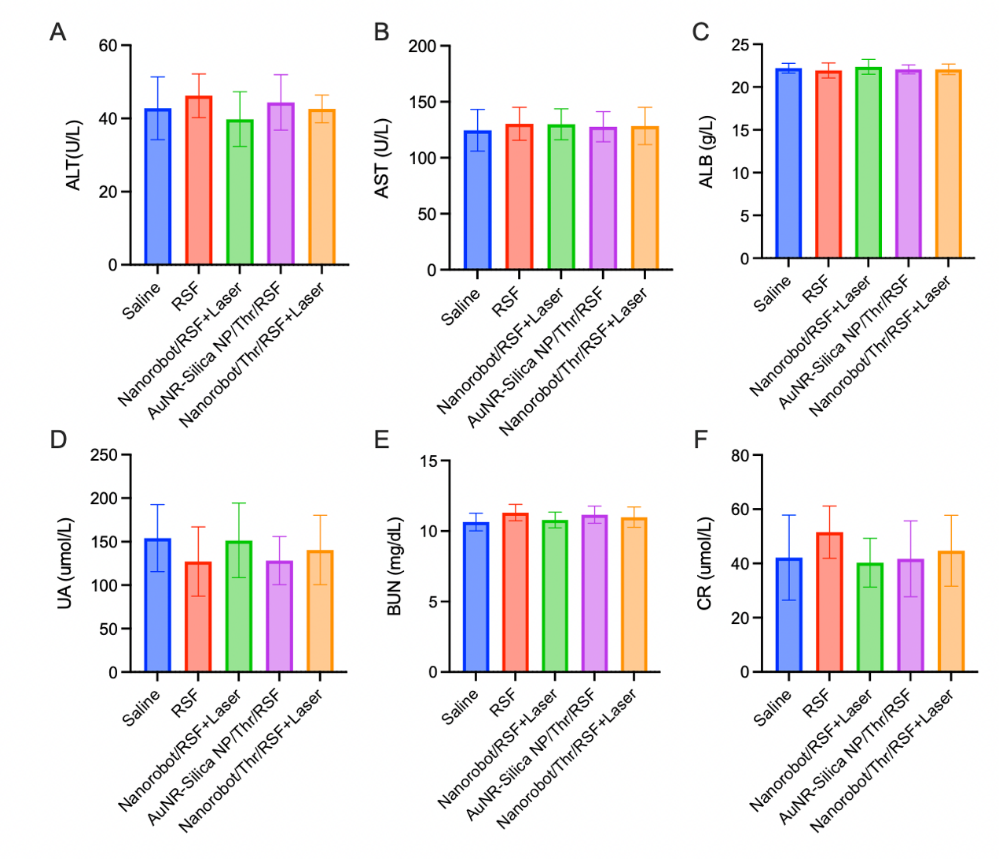


**Fig. S24** Liver function and kidney function after different treatments of HCC spinal metastasis. Alt alanine aminotransferase (ALT), aspartate transaminase (AST), albumin (ALB), uric acid (UA), blood urea nitrogen (BUN), and creatinine (CR)
